# Quantifying ASF Outbreak Dynamics in the Philippines (2019–2023) Using Joinpoint Regression and the Estimated Dissemination Ratio

**DOI:** 10.3390/ani16172811

**Published:** 2026-09-07

**Authors:** Samuel Joseph M. Castro, Roderick T. Salvador, Romeo S. Gundran, Janice S. Garcia, Supitchaya Siriyakhun, Jakkawat Pongsumpan, Kannika Na-Lampang

**Affiliations:** 1 Faculty of Veterinary Medicine, Chiang Mai University, Chiang Mai 50100, Thailand; samcastro_dvm@yahoo.com.ph (S.J.M.C.); siriyakhun.s@gmail.com (S.S.); jakkawat_p@cmu.ac.th (J.P.); 2 College of Veterinary Science and Medicine, Central Luzon State University, Science City of Muñoz 3120, Nueva Ecija, Philippines; roderick.salvador@clsu2.edu.ph (R.T.S.); rsgundran@clsu.edu.ph (R.S.G.); 3 Bureau of Animal Industry, Animal Health and Welfare Division, Quezon City 1128, Metro Manila, Philippines; janice.garcia@bai.gov.ph; 4 Research Center for Veterinary Biosciences and Veterinary Public Health, Faculty of Veterinary Medicine, Chiang Mai University, Chiang Mai 50100, Thailand

**Keywords:** African swine fever (ASF), joinpoint regression, spatiotemporal dynamics

## Abstract

African swine fever (ASF) remains a major constraint on swine production in the Philippines, but interpretation of its national trajectory is complicated by changes in surveillance effort and regional heterogeneity. We analyzed domestic-pig surveillance records from August 2019 through December 2023. After exact-row deduplication and validity screening, 50,852 laboratory records were retained, including 8929 ASF-positive records and 324,741 tested samples. Monthly positive-record counts, annual and surveillance-stream-specific sample positivity, geographic and regional trajectories, year-standardized seasonality indices, Bayesian Information Criterion-selected joinpoint regression, and sliding-window Estimated Dissemination Ratios (EDRs) were evaluated. Positive laboratory records peaked at 733 in September 2020. Annual sample positivity reached 20.6% in 2020 and declined to 3.9% in 2023, despite tested-sample volume increasing from 41,035 to 102,333. Regional peaks were staggered from 2020 through 2023, demonstrating asynchronous detected activity. September and October had the highest mean seasonality indices, although their 95% confidence intervals included 1. The selected joinpoint model identified breakpoints in June, September, and December 2020 and August 2022. A first-order autoregressive correlation structure (AR(1)) sensitivity model supported the 2020 changes but not the August 2022 change. The primary 14-day EDR identified 25 sustained expansion and 28 sustained contraction episodes; 7-day estimates were more volatile, whereas 14- and 21-day estimates were more concordant. These findings indicate a transition from intense nationally detected activity in 2020 to lower sample positivity but persistent, recurrent, and regionally heterogeneous activity through 2023. Integrating record counts, testing denominators, regional patterns, joinpoint regression, and EDR provides a reproducible surveillance framework, but does not estimate population prevalence, causal policy effects, or future transmission.

## Simple Summary

African swine fever (ASF) continues to pose a serious challenge to pig production in the Philippines. This study provides a comprehensive characterization of ASF epidemic dynamics and examines how ASF spread over time and across regions using national laboratory-confirmed surveillance data and complementary temporal analytical methods. The results showed that outbreaks expanded rapidly after 2019, reached a peak in September 2020, and were followed by smaller but recurring waves until 2023. Disease occurrence varied across regions, with earlier activity observed in Luzon and later outbreaks reported in the Visayas and Mindanao. ASF cases were more frequently detected during the later months of the year, particularly from September to November. Over time, the pattern of transmission appeared to shift from large, synchronized outbreaks to smaller, more localized and persistent disease activity. These findings highlight the importance of continuous surveillance, timely outbreak investigation, and adaptable control strategies to support ASF prevention and control in the Philippines.

## 1. Introduction

African swine fever (ASF) is a severe transboundary viral disease of domestic and wild suids that can produce hemorrhagic disease, very high mortality, and major disruption of pig production, trade, and food systems [1,2,3]. Its causative agent, African swine fever virus (ASFV), is a large double-stranded DNA virus that can spread through direct contact, contaminated vehicles and equipment, live-animal movements, and infected pork products [1,2]. The environmental stability of ASFV and the complexity of pig production and marketing networks allow the virus to persist and disseminate over long distances, particularly when detection and movement control are delayed [1,2]. ASF has expanded extensively across Europe and Asia since the introduction of genotype II viruses into Eurasia, producing substantial losses to commercial and smallholder systems and altering regional pork supply [4,5]. Although vaccine development and regulated deployment have advanced, safe and standardized vaccination remains an evolving component of ASF control and does not replace early detection, biosecurity, movement management, traceability, and rapid outbreak response [6,7,8].

The consequences of ASF are especially pronounced in Asian production systems, where pigs support household income, savings, food security, and highly interconnected value chains [4,5]. The Asian epidemic has demonstrated that viral dissemination is shaped not only by biological transmission but also by human behavior, market structure, informal trade, variable biosecurity, and uneven veterinary-service capacity [4,5]. Smallholder and backyard sectors are particularly difficult to protect because production is geographically dispersed, farm-level resources are limited, and pig and pork movements may occur through both formal and informal channels. Under these conditions, surveillance systems must detect rapidly changing epidemic activity while also accounting for substantial heterogeneity in the probability that infection is reported, sampled, and confirmed.

Swine production is an important component of Philippine agriculture and rural livelihoods, and a large proportion of pigs has historically been raised in backyard holdings [9,10,11]. ASF was officially reported in the Philippines in 2019 and subsequently affected backyard and commercial production across Luzon, the Visayas, and Mindanao [11]. The epidemic generated direct animal losses and broader effects on farm income, employment, value-chain activity, pork availability, and household well-being [10,11]. National and local responses progressively included investigation and depopulation, compensation, zoning, movement restrictions, enhanced laboratory surveillance, community-based monitoring, biosecurity programs, and protocols for recovery and repopulation [11]. The archipelagic geography of the country can slow contiguous spread between islands, but it does not prevent discontinuous introductions through movements of live pigs, semen, pork products, vehicles, equipment, and people. Analysis of Philippine shipping permits has identified highly connected trading nodes capable of linking distant production areas, emphasizing the epidemiological importance of movement networks in national preparedness [12].

A growing body of Philippine research has clarified important components of the epidemic. A review on strategies has described diagnostic, biosecurity, surveillance, and governance challenges [11]. Subsequent studies have evaluated risk-based surveillance and farm-level management in Central Luzon [13], transmission-risk profiles in selected provinces [14], and spatiotemporal risk factors among smallholder farms in the Davao Region [15]. Genomic analysis of strains collected from outbreaks between 2021 and 2023 further confirmed the circulation of genotype II viruses across the three major island groups [16]. These studies provide essential spatial, operational, and molecular evidence, but they address different geographic areas, time periods, surveillance populations, and epidemiological questions.

National program data also create important interpretive challenges. Counts of positive laboratory records are influenced by the amount of testing, the geographic and production sectors sampled, the operational purpose of surveillance, diagnostic access, and reporting behavior. Risk-based surveillance intentionally concentrates effort in higher-risk populations and therefore cannot be interpreted as a probability sample of the national pig population [17]. Imperfect and heterogeneous detection can likewise produce false zeros, delay recognition of epidemic change, and distort comparisons among places or periods [18]. Consequently, a decline in positive-record counts does not necessarily indicate a proportional decline in infection, whereas an increase in detected records may arise from broader surveillance even when the proportion of tested samples that is positive decreases. National ASF dynamics are therefore more defensibly evaluated by combining record-based epidemic curves with testing denominators, sample positivity, surveillance-stream composition, regional trajectories, and explicit assessment of temporal uncertainty.

Time-series methods can extend descriptive surveillance by distinguishing broad structural changes from short-term fluctuations. Segmented or joinpoint regression estimates changes in slope and identifies candidate breakpoints in an observed temporal series [19,20]. However, breakpoint estimates from surveillance counts must be evaluated together with residual diagnostics and denominator information because autocorrelation, non-constant variance, and changes in reporting effort can affect inference [18,19]. The Estimated Dissemination Ratio (EDR) provides a complementary measure by comparing detected events in a recent time window with those in the immediately preceding window [21]. EDR is transparent and operationally useful for identifying periods of expansion and contraction, but it is not equivalent to a reproduction number and its performance depends on the chosen window length and the density of the underlying surveillance series.

Despite the national importance of ASF, few studies have integrated the full range of surveillance information needed to interpret how detected activity changed across the Philippines after the initial epidemic phase. In particular, national analyses have not jointly examined laboratory-record counts, tested-sample volume, sample positivity, surveillance-stream heterogeneity, regional temporal trajectories, seasonality with uncertainty, structural trend changes, short-term dissemination episodes, and data completeness within a single reproducible framework. To our knowledge, EDR has not previously been applied to national ASF laboratory surveillance in the Philippines. Addressing these gaps is important because a national aggregate may conceal simultaneous regional expansion and contraction, and apparent trends may reflect both epidemic processes and changes in surveillance.

Accordingly, this study used national domestic-pig surveillance data from August 2019 through December 2023 to:

(1) Audit and describe the laboratory surveillance dataset and quantify tested-sample volume and sample positivity overall and by year, region, and surveillance stream;

(2) Characterize national and regional spatiotemporal patterns and assess monthly seasonality;

(3) Identify structural changes in national monthly positive laboratory-record counts using joinpoint regression and evaluate model diagnostics and an autoregressive sensitivity specification;

(4) Quantify short-term dissemination using 7-, 14-, and 21-day EDR windows, sustained expansion and contraction episodes, and regional EDR completeness; and

(5) Examine the temporal correspondence between major national control measures and the observed surveillance indicators descriptively.

The study was designed to characterize detected surveillance dynamics rather than estimate population prevalence, causal policy effects, or future transmission.

## 2. Materials and Methods

### 2.1. Study Design, Setting, and Data Source

A retrospective observational study was conducted using the national ASF surveillance database for the Philippines. The primary analytic period extended from 8 August 2019 through 31 December 2023. The analysis concerned domestic-pig surveillance records; the database did not contain a dedicated wild-suid surveillance stream.

The source database was maintained under the national ASF surveillance program of the Bureau of Animal Industry and contained laboratory accession information, a combined date-of-onset/date-of-sample-collection field, administrative location, geographic coordinates, sample and test information, surveillance purpose, testing facility, qualitative result, sample-count fields, farm population, and farm type. Because onset and sample collection were not stored as separate variables, the recorded date was used as the analytic event date and was not interpreted uniformly as the true date of clinical onset or infection.

Testing was performed through the Animal Disease Diagnosis and Reference Laboratory and Regional Animal Disease Diagnostic Laboratories. Database assay labels were predominantly real-time polymerase chain reaction, with conventional PCR, insulated isothermal PCR, and other minor laboratory methods also represented. The final qualitative result supplied in the surveillance database was used to classify each record as Positive, Negative, or Not Tested. Assay-specific cycle-threshold values and confirmatory algorithms were unavailable and were not reinterpreted.

### 2.2. Data Processing and Eligibility

**Figure 1:**
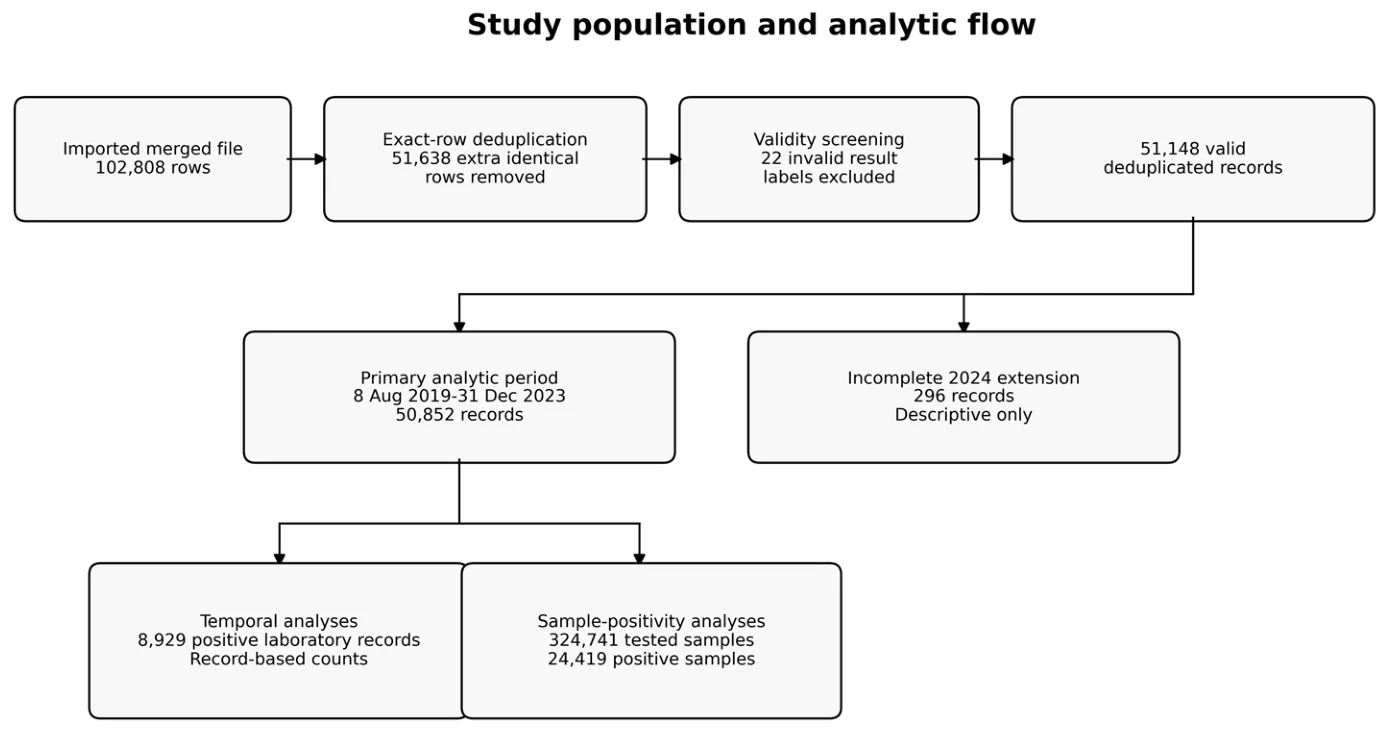
Study population and analytic flow. The 51,148 valid deduplicated records were partitioned into the primary 2019–2023 analytic dataset and the incomplete 2024 descriptive extension.

The merged export contained 102,808 rows. After removal of exact duplicates (rows identical across all 19 source fields) and 22 records with invalid result labels, 51,148 valid deduplicated records remained. These were separated into the primary 2019–2023 analytic dataset (50,852 records) and an incomplete 2024 extension (296 records), which was analyzed descriptively and excluded from the primary temporal models. The analytic flow is summarized in Figure 1, and full record-level eligibility and quality checks are reported in Supplementary Table S1.

Administrative-region and surveillance-purpose labels were standardized before analysis. When a negative-sample count was missing but could be reconstructed unambiguously from the recorded total, positive, and untested components, the reconstructed value was used. Records without a valid positive-plus-negative sample denominator were excluded from sample-positivity calculations but remained eligible for record-based analyses when the final qualitative result was valid.

### 2.3. Analytic Units, Surveillance Streams, and Sample Positivity

A laboratory record was defined as one exact-row-deduplicated database row. A positive laboratory record was a row with a final qualitative result of Positive. Each positive record contributed one count to temporal analyses, regardless of the number of samples represented in that row. The same record-based definition was applied throughout the study period.

The database contained no stable unique premises identifier. Consequently, a laboratory record could not be assumed to represent a unique pig, farm, household, premises, village, or epidemiologically linked outbreak. Positive- and negative-sample totals were used only for sample-volume and positivity analyses. Untested samples were excluded from the denominator. Sample positivity was calculated using Equation (1).

(1) $$P_{ASF}\;(\%)=100\times \frac{\sum \;P_{i}}{\sum \;(P_{i}+N_{i})}$$

where:

$P_{ASF}$ denotes sample positivity expressed as a percentage;
$P_{i}$ and $N_{i}$ are the numbers of ASF-positive and ASF-negative samples, respectively, in eligible laboratory record *i*; and
Σ indicates summation across all eligible records.

Only records with a valid positive-plus-negative sample denominator were included. Untested samples were excluded from both the numerator and denominator.

Surveillance purpose was harmonized into four principal streams: routine surveillance, event-driven disease investigation, repopulation screening, and local-shipment testing. Records with missing or nonstandard surveillance-purpose labels were included in the overall analyses if they met all other eligibility criteria, but they were excluded from analyses comparing the four principal surveillance streams.

These streams differed in purpose and trigger. Routine surveillance applied risk- and area-based testing initiated by program criteria rather than by clinical signs; event-driven disease investigation was reactive, triggered by reports of clinical disease or unusual mortality; and repopulation screening and local-shipment testing sampled predominantly apparently healthy animals to support recovery and movement clearance. Testing used predominantly real-time PCR at the national reference and regional diagnostic laboratories. Because event-driven disease investigation contributed most positive records, the positive-record series should be interpreted as reflecting a predominantly suspicion-driven, reactive surveillance system rather than systematic probability-based sampling of the pig population, and stream composition was not held constant as the national program expanded.

### 2.4. Descriptive Spatiotemporal Analysis

Laboratory-record counts, tested-sample totals, positive-sample totals, and sample positivity were summarized overall and by calendar year, administrative region, surveillance stream, and farm type. The 2019 estimates were treated as partial-year values.

National and regional epidemic curves were generated from calendar-month positive-record counts. For each administrative region, the first and last recorded positive month, peak month, peak monthly count, and cumulative positive-record count were summarized. The first recorded positive month was interpreted as the first detection represented in the database and not necessarily as the true date of viral introduction.

Annual geographic maps displayed all geocoded surveillance records faintly and overlaid positive laboratory records. Point locations represented the coordinates recorded in the surveillance database and were not interpreted as unique premises. A regional heatmap used a pseudo-logarithmic color transformation to preserve visibility across low- and high-count region-months. Common-scale and region-specific regional curves were prepared as supplementary figures.

### 2.5. Seasonality Analysis

National seasonality was evaluated using the four complete calendar years, 2020–2023. For calendar month m in year y, the year-standardized seasonality index was calculated using Equation (2).

(2) $${SI}_{ym}=\frac{C_{ym}}{{\bar{C}}_{y}},\;{\bar{C}}_{y}=\frac{1}{12}\;\sum_{m=1}^{12}\;C_{ym}$$

where:

${SI}_{ym}$ is the seasonality index for month m of year y;
$C_{ym}$ is the number of positive laboratory records in that month;
${\bar{C}}_{y}$ is the mean monthly positive-record count for year y; m ranges from January (1) to December (12).

An index of 1 indicates that the month equaled the corresponding year’s mean monthly count; values above or below 1 indicate higher or lower activity, respectively.

The four annual indices were averaged for each calendar month, and t-based 95% confidence intervals were calculated from between-year variability. Because only four complete years contributed and 12 months were examined, the month-specific estimates were interpreted descriptively and no multiple-comparison-adjusted seasonal significance claims were made.

Regional seasonality was exploratory. The same within-year standardization was applied within region-year strata. Region-years with no positive records produced undefined indices, and regional month-specific means were displayed only when at least two region-years contributed.

### 2.6. Joinpoint Regression Analysis

Structural changes in the national monthly positive-record series were assessed using continuous piecewise-linear regression. The joinpoint model was specified as Equation (3).

(3) $$Y(t)=\beta_{0}+\beta_{1}t+\sum_{j=1}^{k}\;\delta_{j}[t-\psi_{j}{]}_{+}+\varepsilon (t)$$

where:

*Y_(t)_* = the national count of positive laboratory records in month t;
*β*_0_ = the intercept;
*β*_1_ = the baseline monthly slope;
*k* = the number of joinpoints;
*ψ_j_* = the location of joinpoint j;
*δ_j_* = the change in slope after joinpoint j;
*ε_t_* = the residual error for month t.

Models containing zero through five joinpoints were considered. Candidate breakpoint locations were initialized using segmented regression and refined to calendar-month boundaries. Adjacent breakpoints and study endpoints were required to be separated by at least three monthly observations. Candidate models were fitted by least squares and compared using the Bayesian Information Criterion (BIC), calculated using Equation (4); the model with the minimum BIC was selected [22].

(4) $$BIC=n\;ln(\frac{RSS}{n})+p\;ln(n)$$

where:

*n* = the number of monthly observations;
*RSS* = the residual sum of squares from the candidate model;
*p* = the total number of estimated parameters, including regression coefficients and breakpoint locations; and
ln = the natural logarithm.

Lower BIC values indicate a preferable balance between model fit and complexity.

Segment slopes were reported as the estimated monthly change in positive laboratory records, with conditional 95% confidence intervals given the selected breakpoints. Model adequacy was evaluated using residual-versus-fitted and normal Q–Q plots and a lag-12 Ljung–Box test. Because surveillance time series commonly exhibit serial dependence, the selected breakpoint structure was refitted using generalized least squares with an AR(1) residual correlation structure as a sensitivity analysis [19].

### 2.7. Estimated Dissemination Ratio (EDR)

A continuous daily series was constructed from positive laboratory-record counts, assigning zero to dates with no positive records. For a window length w, the Estimated Dissemination Ratio (EDR) compared the total in the most recent w days with the total in the immediately preceding w days, as specified in Equation (5) [21].

(5) $${EDR}_{t}(w)=\frac{\sum_{i=t-w+1}^{t}\;x_{i}}{\sum_{i=t-2w+1}^{t-w}\;x_{i}}$$

where:

*EDR_t_*(*w*) = the EDR calculated for day t using a window of w days;
*x_i_* = the number of positive laboratory records on day *i*;
the numerator is the total during the most recent *w* days (*t* − *w* + 1 through *t*);
the denominator is the total during the immediately preceding *w* days (*t* − 2*w* + 1 through *t* − *w*).

EDR > 1 denotes expansion in detected records, EDR < 1 denotes contraction, and EDR is undefined when the preceding-window total is zero.

Within each estimate, the numerator and denominator windows were adjacent and non-overlapping; successive estimates advanced by one day and therefore formed a sliding series. A 14-day window was selected a priori as a pragmatic surveillance interval that spans the multi-day clinical and detection process of ASF while reducing instability from daily reporting and investigation clusters [1,2]. It was not assumed to represent a fixed biological generation interval. Sensitivity analyses used 7- and 21-day windows.

Sustained expansion and contraction episodes were defined as at least 14 consecutive days with valid EDR values above or below 1, respectively. Episode start and end dates, duration, median EDR, extreme EDR, and positive-record totals were summarized. Window specifications were compared using descriptive summaries, paired-day Pearson and Spearman correlations, and agreement in expansion-versus-contraction classification. Regional 14-day EDR series were used principally to quantify the proportion of evaluated days for which an EDR could not be calculated.

### 2.8. Descriptive Correspondence with Control Measures

A chronology of major national ASF control measures issued from 2019 through 2023 was compiled from primary government documents. The chronology covered emergency investigation and depopulation, compensation, zoning and movement controls, national coordination, community-based surveillance, recovery and zone progression, biosecurity and diagnostic standards, sentinel procedures, and movement certification.

Policy dates were overlaid descriptively on the national epidemic curve and summarized alongside annual sample positivity, selected joinpoints, and annual counts of sustained EDR episodes. No interrupted time-series effect, counterfactual comparison, lagged intervention effect, or causal policy-effectiveness estimate was calculated.

### 2.9. Statistical Software and Reproducibility

All analyses were performed in R version 4.6 using fully reproducible scripts. Data preparation and visualization were conducted using the tidyverse, lubridate, janitor, ggplot2, patchwork, ggrepel, sf, and rnaturalearth packages. Candidate joinpoints were initialized using segmented. The AR(1) generalized least-squares sensitivity model was fitted using nlme. The rolling Estimated Dissemination Ratio calculations were performed using zoo.

### 2.10. Ethical Considerations

This study used secondary surveillance data collected as part of routine animal health monitoring. The analytical dataset used for publication did not contain personal, owner, or farm identifiers. National ASF surveillance data was provided by the National African Swine Fever Prevention and Control Program (NASFPCP) with a required Data Request Form and corresponding Memorandum of Approval of the Director of the Bureau of Animal Industry (BAI).

## 3. Results

### 3.1. Analytic Dataset

After exact-row deduplication and validity screening (Section 2.2; Supplementary Table S1), the primary analytic dataset comprised 50,852 laboratory records collected from 8 August 2019 through 31 December 2023.

The primary dataset included 8929 ASF-positive laboratory records and 324,741 tested samples, comprising 24,419 positive and 300,322 negative samples. Overall sample positivity was 7.52%. Temporal analyses counted each deduplicated positive laboratory record once, whereas sample-positivity analyses used the corresponding positive- and negative-sample totals.

### 3.2. Surveillance Composition

Routine surveillance accounted for 31,846 laboratory records, followed by disease investigation (8854), repopulation screening (5304), and local-shipment testing (4846). Positivity varied markedly by surveillance stream (Table 1). Disease investigation accounted for 6512 positive laboratory records and 17,512 positive samples, with a sample positivity of 63.2%. Routine surveillance had 2192 positive records and a positivity of 4.3%. Local-shipment testing and repopulation screening had substantially lower positivity, at 1.1% and 0.6%, respectively.

### 3.3. National Temporal Distribution and Annual Positivity

**Figure 2:**
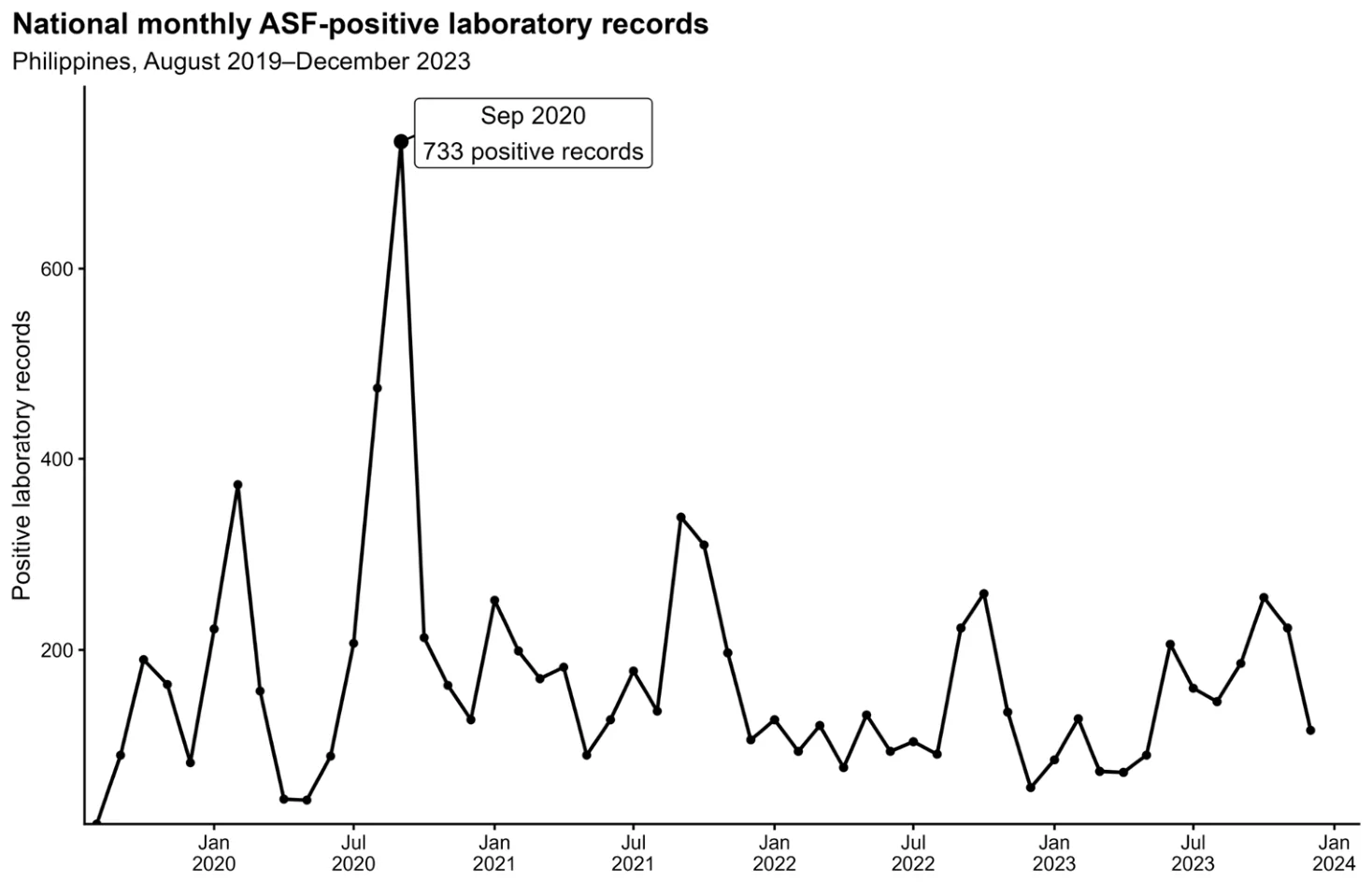
National monthly ASF-positive laboratory records, Philippines, August 2019–December 2023. Each deduplicated database row with a qualitative positive result contributed one laboratory record, irrespective of the number of positive samples summarized in the row.

The monthly national series increased after the first recorded detections in August 2019 and reached its maximum in September 2020, with 733 positive laboratory records (Figure 2). The next highest monthly totals were recorded in August 2020 (475), February 2020 (373), September 2021 (339), October 2021 (310), October 2022 (259), and October 2023 (255). Positive records remained present throughout the remainder of the primary study period.

**Figure 3:**
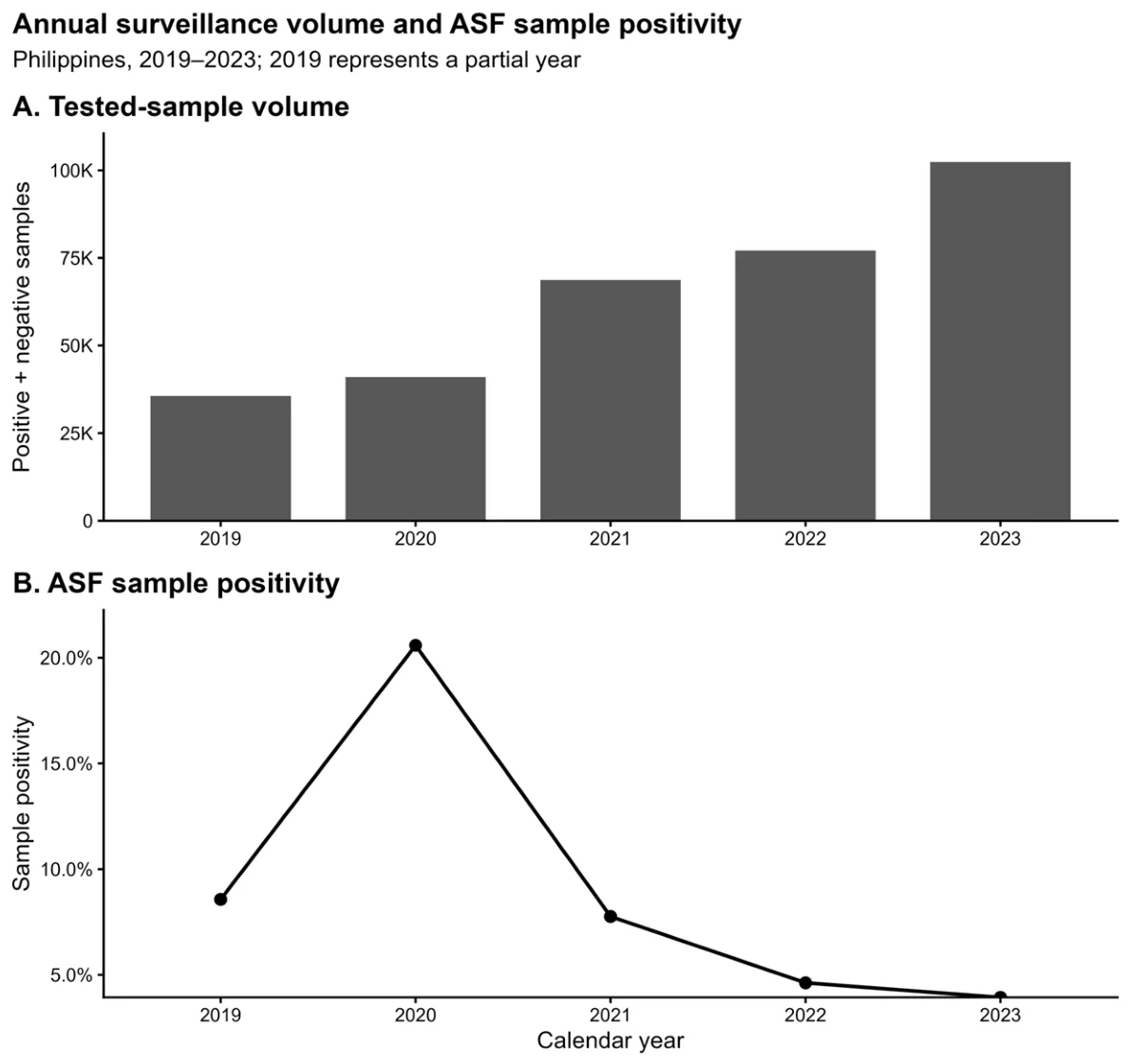
Annual surveillance volume and ASF sample positivity, Philippines, 2019–2023. Tested-sample volume is the sum of positive and negative samples. The 2019 values represent a partial year beginning on 8 August.

Annual positive-record totals were 544 during the partial 2019 observation period, 2846 in 2020, 2286 in 2021, 1513 in 2022, and 1740 in 2023. Annual tested-sample volume increased substantially during the study period, while sample positivity was highest in 2020 and declined thereafter (Table 2; Figure 3). During the partial 2019 period, 3048 of 35,569 tested samples were positive (8.6%). Positivity increased to 20.6% in 2020 (8448/41,035) and subsequently declined to 7.8% in 2021, 4.6% in 2022, and 3.9% in 2023. Routine-surveillance positivity showed the same broad pattern.

### 3.4. Geographic Distribution and Regional Epidemic Trajectories

**Figure 4:**
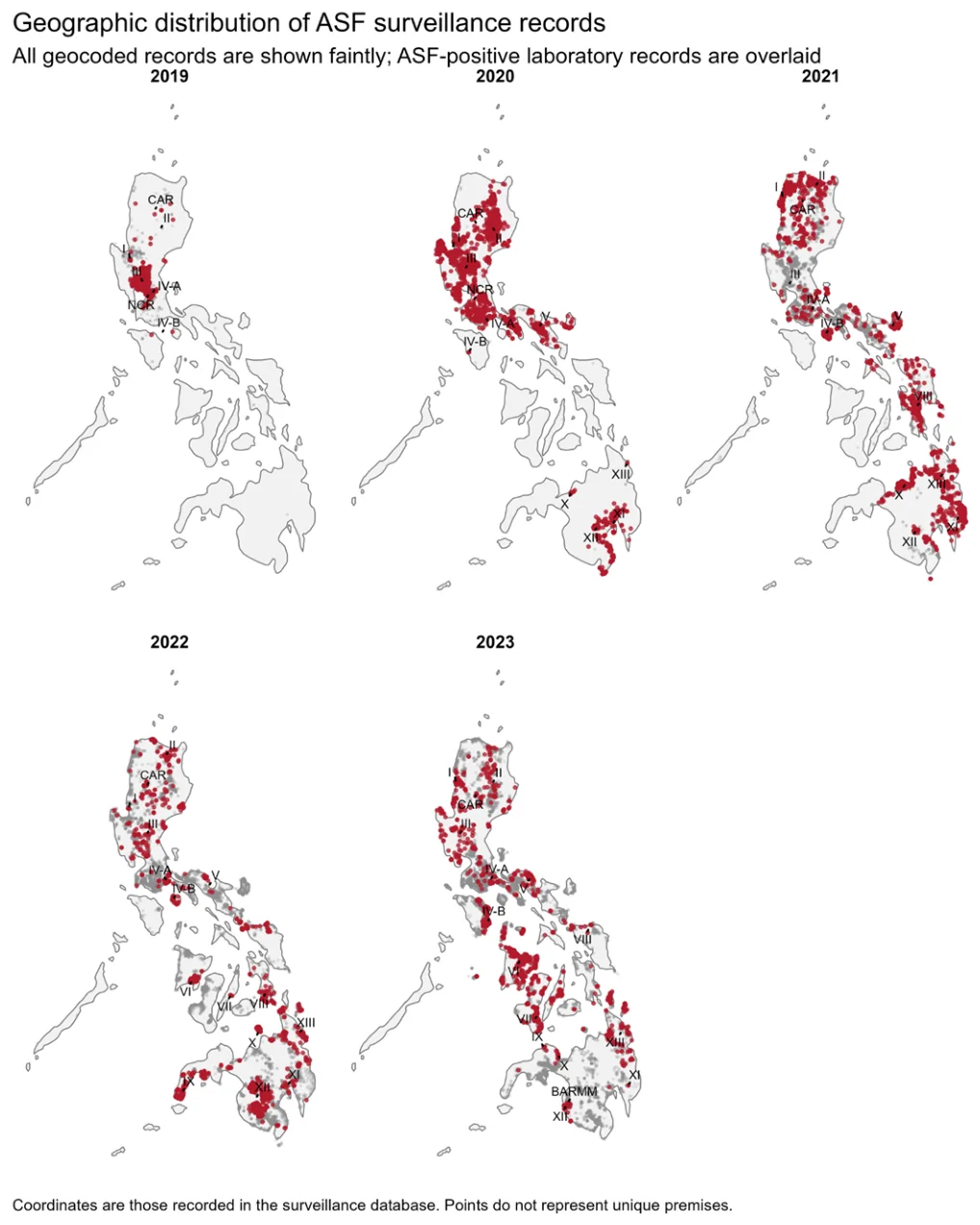
Geographic distribution of ASF surveillance records, Philippines, 2019–2023. All geocoded records are shown faintly and ASF-positive laboratory records are overlaid. Points represent recorded coordinates and do not represent unique premises.

**Figure 5:**
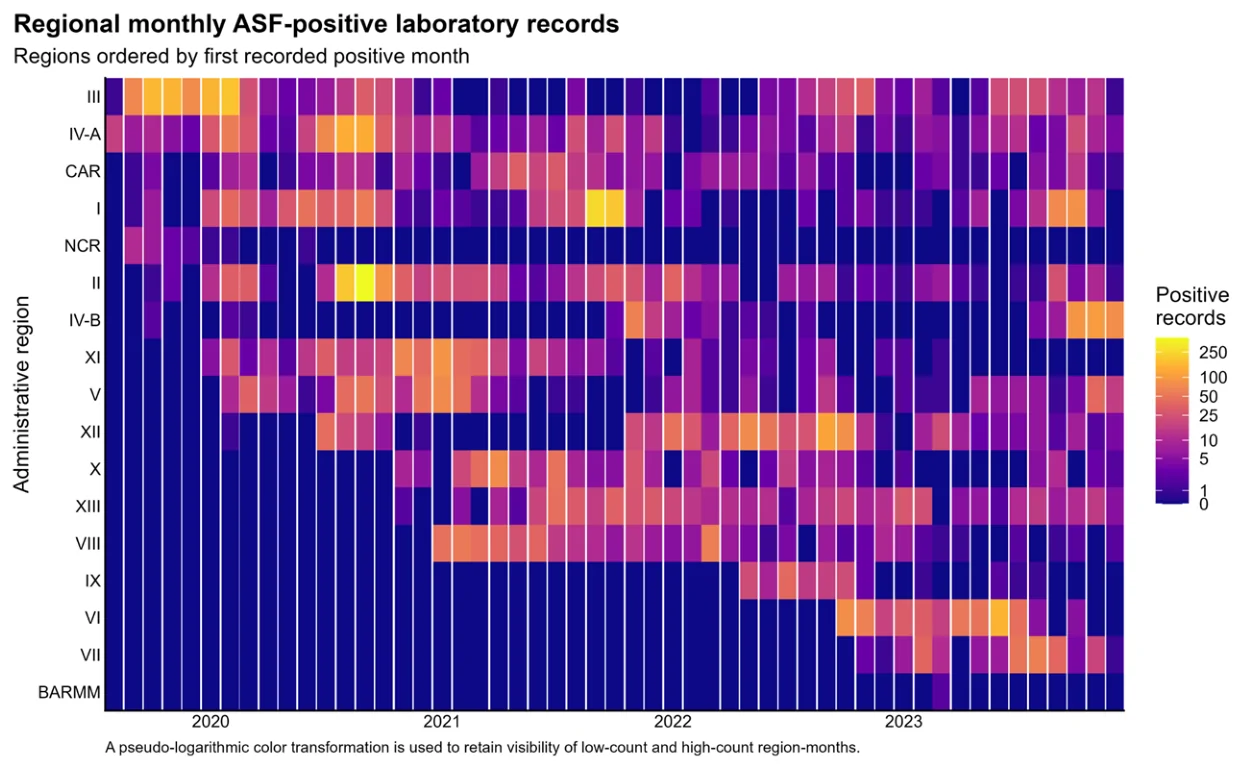
Regional monthly ASF-positive laboratory records, Philippines, August 2019–December 2023. Regions are ordered by first recorded positive month. A pseudo-logarithmic color scale retains visibility of both low-count and high-count region-months.

The annual maps showed expansion in the geographic distribution of positive laboratory records from early concentrations in Luzon to detections across the major island groups (Figure 4). The regional heatmap showed substantial differences in the timing, magnitude, and duration of detected activity (Figure 5; Supplementary Figures S1 and S2). Regions III (Central Luzon) and IV-A (CALABARZON) had the earliest positive months, in August 2019. Later first detections occurred progressively across Luzon, Mindanao, and the Visayas, with Region VI (Western Visayas) first represented in October 2022, Region VII (Central Visayas) in November 2022, and BARMM (Bangsamoro Autonomous Region in Muslim Mindanao) in March 2023.

Peak timing was similarly heterogeneous. Region III peaked in February 2020 with 193 positive records; Region IV-A in August 2020 with 136; and Region II (Cagayan Valley) in September 2020 with 419. Later regional peaks occurred in Region I (Ilocos Region) in September 2021 (260), Region XII (SOCCSKSARGEN) in September 2022 (119), Region VI in June 2023 (151), Region VII in August 2023 (60), and Region IV-B (MIMAROPA) in November 2023 (104). The largest cumulative regional totals were recorded in Region II (1188), Region III (1156), and Region I (1067) (Supplementary Table S2).

Regional sample positivity ranged from 0.6% in BARMM to 27.2% in Region II. The highest values were recorded in Region II (27.2%), Region VIII (17.9%), CAR (16.4%), Region I (16.3%), and Region XIII (15.5%). The lowest values were recorded in BARMM (0.6%), Region IV-A (3.5%), Region XII (3.9%), Region X (4.4%), and Region IV-B (5.3%) (Supplementary Table S3 and Figure S3).

### 3.5. Seasonal Distribution of Positive Laboratory Records

**Figure 6:**
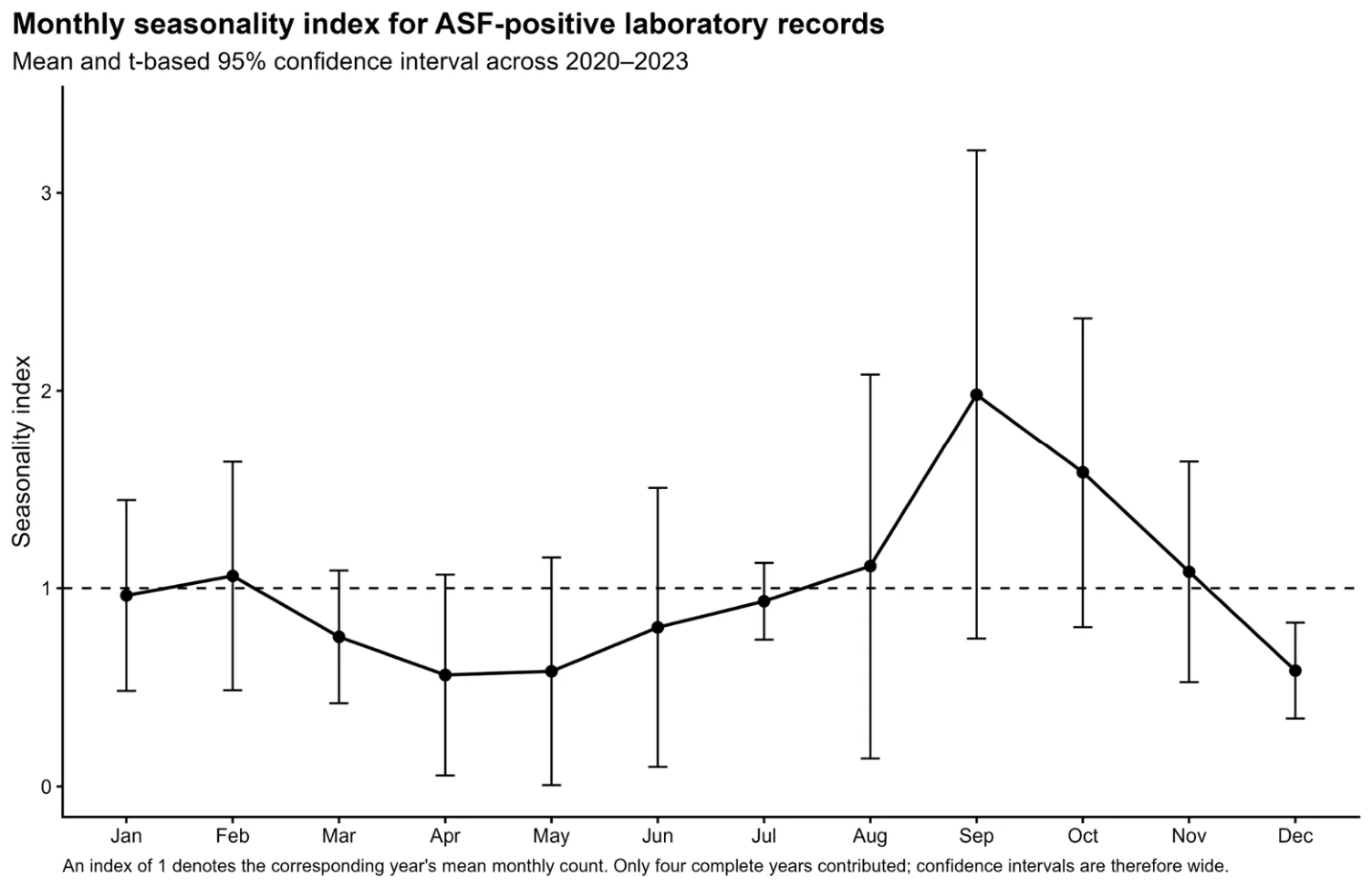
Mean monthly seasonality index for ASF-positive laboratory records, Philippines, 2020–2023. Points show means across four complete calendar years, error bars show t-based 95% confidence intervals, and the dashed line denotes an index of 1.

Across the four complete calendar years, September had the highest mean seasonality index (1.98), followed by October (1.58), August (1.11), November (1.08), and February (1.06) (Figure 6; Supplementary Table S4). The lowest mean indices occurred in April (0.56), May (0.58), and December (0.58). Between-year uncertainty was substantial. The 95% confidence intervals were 0.75–3.21 for September and 0.80–2.37 for October, and both included the reference value of 1. December had a mean index of 0.58 with a 95% confidence interval of 0.34–0.83. The exploratory regional analysis showed heterogeneous month-specific patterns (Supplementary Figure S4).

### 3.6. Joinpoint Regression

**Figure 7:**
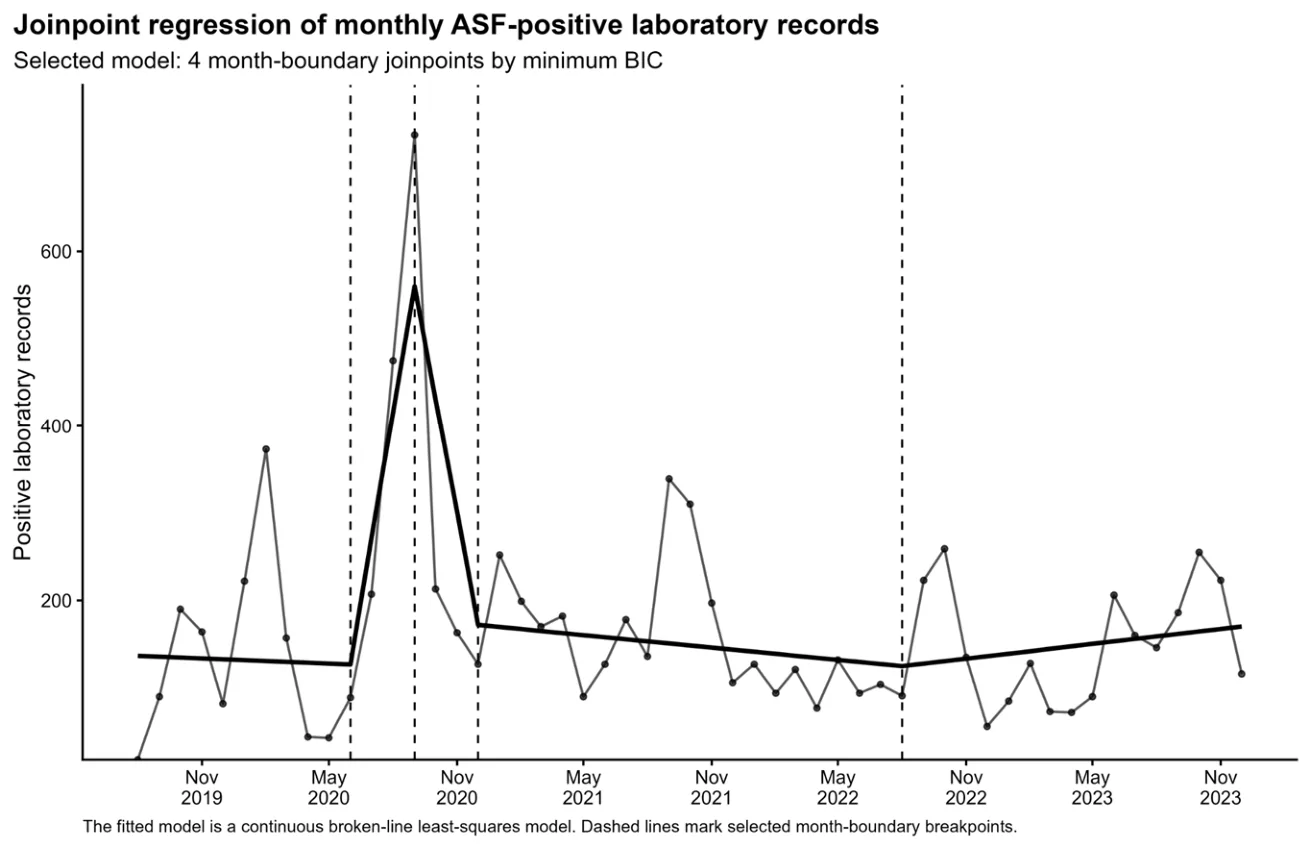
Joinpoint regression of monthly ASF-positive laboratory records, Philippines, August 2019–December 2023. Dashed lines mark the four BIC-selected month-boundary breakpoints; the heavy solid line shows the continuous broken-line least-squares fit.

Among candidate models containing zero through five joinpoints, the four-joinpoint model had the lowest BIC (508.62), compared with 511.06 for the model without joinpoints. The selected breakpoints occurred at June 2020, September 2020, December 2020, and August 2022 (Figure 7; Supplementary Table S5).

The segment from August 2019 through June 2020 showed no clear linear change. The fitted count then increased from June to September 2020 by 144.40 records/month (95% CI, 88.40 to 200.41), followed by a decline from September to December 2020 of −129.31 records/month (95% CI, −179.35 to −79.28). Neither the December 2020–August 2022 segment nor the August 2022–December 2023 segment showed a clear linear change (Table 3).

The Ljung–Box test identified residual temporal autocorrelation (Q = 17.63, df = 7, *p* = 0.014). Normal Q–Q and residual-versus-fitted plots also showed departures from normality and constant variance (Supplementary Figures S6 and S7). In the AR(1) generalized least-squares sensitivity model, change-in-slope terms at the June, September, and December 2020 breakpoints remained statistically supported, whereas the August 2022 change-in-slope term was not supported (estimate = 3.96, *p* = 0.595).

### 3.7. Estimated Dissemination Ratio

**Figure 8:**
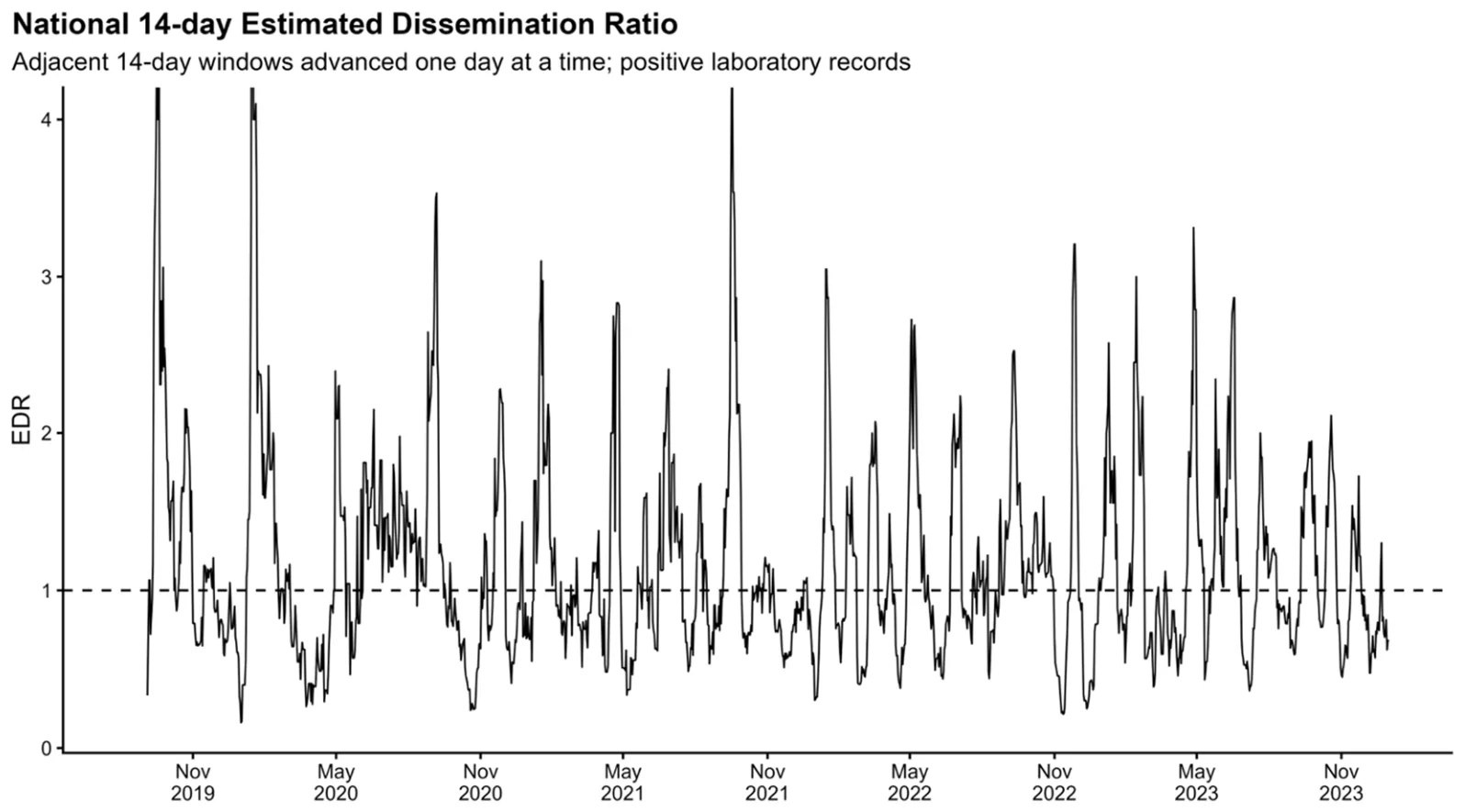
National 14-day Estimated Dissemination Ratio for ASF-positive laboratory records, Philippines, August 2019–December 2023. Values above 1 indicate more positive records in the most recent 14 days than in the immediately preceding 14 days. The displayed y-axis is truncated at the 99.5th percentile; full numerical values were retained in the analysis tables.

The national 14-day EDR was calculable on all 1580 evaluated days after the initial 27-day period required to form two complete windows. The median EDR was 0.98 and the mean was 1.17 (Figure 8; Table 4). The maximum EDR was 7.80 on 18 September 2019, and the minimum was 0.16 on 1 January 2020.

Using the predefined minimum duration of 14 consecutive days, 25 sustained expansion episodes and 28 sustained contraction episodes were identified. The expansion episode containing the largest number of positive records occurred from 13 August to 14 September 2020; it lasted 33 days, contained 755 positive records, and had a median EDR of 1.34 and a peak EDR of 3.53. The longest expansion occurred from 5 June to 11 August 2020 (68 days; 365 records). Other major episodes occurred during January–February 2020, September 2021, October 2022, and May–June and September–October 2023 (Table 5; Supplementary Table S7).

The 7-day EDR series was substantially more volatile than the longer-window specifications (Supplementary Figure S8). The 14- and 21-day series had a Pearson correlation of 0.52, a Spearman correlation of 0.60, and 73.1% agreement in expansion-versus-contraction classification (Supplementary Table S6). Regional EDR completeness varied substantially, with undefined-day percentages ranging from 18.4% in Region IV-A to 99.1% in BARMM (Supplementary Table S3).

## 4. Discussion

This national analysis indicates that detected ASF activity in the Philippines evolved through an intense expansion phase centered on 2020, followed by a period of lower sample positivity but persistent and geographically heterogeneous detection through 2023. This interpretation is supported by the convergence of several indicators rather than by laboratory-record counts alone. The September 2020 national maximum coincided with the highest annual sample positivity and the strongest joinpoint-defined increase. Meanwhile, later years combined substantially greater testing volume with lower positivity, flatter long-term slopes, and recurrent short-term EDR expansion episodes. Regional trajectories were markedly asynchronous, with early peaks in several Luzon regions and later peaks in parts of Mindanao, and the Visayas. Taken together, these findings are more consistent with a transition from broad national amplification toward persistent localized activity than with either uninterrupted national decline or repeated nationwide waves.

### 4.1. The National Trajectory and Surveillance Intensity

The 2020 peak represents the clearest period of nationally detected epidemic expansion. Positive laboratory records reached their monthly maximum in September 2020. Annual positivity reached 20.6%, and the selected joinpoint model identified a steep increase from June to September followed by a steep decline through December. The concurrence of a high record count and a high proportion of positive samples argue against an explanation based only on expanded testing. Instead, it indicates that the mid-2020 increase involved both more testing activity and a greater concentration of positive samples among those tested. This pattern is compatible with the early phase of a major transboundary disease epidemic, when investigations are focused on clinically affected populations and recently invaded areas. However, it should still be described as a peak in detected activity rather than the exact biological peak of infection.

The later trajectory requires a different interpretation. Tested-sample volume increased from 41,035 in 2020 to 102,333 in 2023, while sample positivity declined from 20.6% to 3.9%. Routine-surveillance positivity showed the same broad decline, reducing the likelihood that the national pattern was generated solely by the increasing contribution of movement or repopulation testing. In veterinary surveillance, however, the number of detected events is a joint product of disease occurrence, sampling intensity, selection of the tested population, diagnostic access, and reporting behavior [17,18]. The opposing movement of testing volume and positivity therefore supports a genuine reduction in the proportion positive among tested samples after 2020. Nonetheless, it does not establish a population-level decline in incidence or prevalence.

This distinction is particularly important for interpreting 2023. Positive laboratory records increased from 1513 in 2022 to 1740 in 2023, even as sample positivity declined from 4.6% to 3.9%. An expanded surveillance system can detect more absolute positives while producing a lower positivity rate because the denominator increasingly includes lower-risk populations and a wider geographic area. Similar surveillance effects have been documented when active searching or improved ascertainment increased recorded disease events independently of a comparable increase in underlying population risk [18,23]. The 2023 pattern is therefore best interpreted as the combination of wider surveillance coverage and continuing localized ASF activity, rather than as evidence of a second national epidemic wave.

Programmatic sample positivity should consequently be treated as a surveillance outcome, not as a national prevalence estimate. Risk-based surveillance intentionally concentrates effort in groups with higher expected risk, improving detection efficiency but limiting direct extrapolation to the source population [17]. Structured surveys in other Southeast Asian settings have shown that estimates derived from defined sampling frames and opportunistic laboratory submissions answer different epidemiological questions [23,24]. In the present study, the absence of a national pig population denominator, farm-level time at risk, and a probability sampling design precludes estimation of incidence or prevalence. The annual positivity series is nevertheless valuable because it provides a denominator-adjusted description of the tested population and prevents changes in raw record counts from being interpreted in isolation.

### 4.2. Surveillance-Stream Heterogeneity

The large differences among surveillance streams demonstrate that surveillance purpose is a major determinant of aggregate positivity. Disease investigation had 63.2% sample positivity, whereas routine surveillance, local-shipment testing, and repopulation screening had positivities of 4.3%, 1.1%, and 0.6%, respectively. These contrasts are expected because the streams sampled populations with very different pre-test probabilities. Event-driven disease investigation preferentially included animals or holdings in which ASF was already suspected. Movement and repopulation testing, by contrast, were designed largely to support clearance, safe movement, recovery, or demonstration of freedom and therefore included many apparently healthy or lower-risk animals. High positivity in disease investigation should therefore be understood as enrichment of the tested population, not as evidence that this stream was more effective than the others.

This finding has two broader implications. First, national positivity can change when the relative contribution of surveillance streams changes, even if risk within each stream is stable. Stratifying positivity by purpose is therefore essential for interpreting routine surveillance databases. Second, low positivity in movement or repopulation screening is not evidence that these activities are unimportant. Similarly, movement testing provides a preventive barrier at points where infected pigs or products could connect otherwise separated production areas.

Despite broad surveillance coverage, undetected infection remains plausible. Imperfect and heterogeneous detection can distort observed disease distributions and risk estimates, particularly when the probability of reporting or testing varies among regions and production systems [18]. The present database combined active programmatic streams with event-driven disease investigation, and the analytic date represented the recorded collection or detection date rather than the date of infection or clinical onset. Incubation, farmer recognition, notification, sample transport, laboratory processing, and database entry all introduce variable delays. Consequently, a region’s first positive record should be interpreted as its first recorded detection in this database, not as the exact date of viral introduction.

Reporting behavior may also have changed over time. In smallholder systems, the anticipated economic consequences of quarantine, depopulation, movement restriction, and production loss can discourage prompt notification, particularly when compensation is viewed as inadequate or delayed [10,25]. Conversely, greater awareness, community-based surveillance, improved laboratory access, and confidence that reporting will lead to support may increase ascertainment. These opposing forces make the direction of reporting bias difficult to predict. The recorded positive laboratory records should therefore be considered a lower bound on detected occurrence rather than a complete census of infected pigs, premises, or epidemiologically linked outbreaks.

### 4.3. Regional Asynchrony and Dissemination

The regional findings show that the national curve was an aggregate of epidemics occurring at different stages. Several Luzon regions were represented early and reached major peaks during 2020 or 2021, whereas Region XII peaked in 2022 and Regions VI, VII, and IV-B showed prominent peaks in 2023. Aggregation at the national level can therefore conceal simultaneous regional expansion, contraction, first emergence, and recurrent activity. This helps explain why a broadly lower national positivity after 2020 coexisted with sustained EDR expansions and substantial later peaks in individual regions.

The archipelagic setting may slow continuous geographic spread while still permitting discontinuous introductions. Sea boundaries separate many pig-producing populations, but movement of live pigs, semen, pork products, vehicles, equipment, and people can bridge these barriers. Analysis of legal shipping permits in the Philippines identified highly connected trading nodes and average movement distances exceeding 100 km for live pigs, demonstrating the capacity of formal trade to connect distant production areas [12]. International evidence similarly identifies human-mediated trade and travel as major drivers of long-distance dissemination of pig diseases [26]. Legal permit data do not capture informal or undocumented movements, so the true network may be broader and more difficult to regulate.

The available surveillance data cannot determine whether later regional peaks resulted from previously undetected circulation, persistence within local production networks, or repeated reintroduction. Distinguishing these processes requires unique premises identifiers, movement histories, outbreak linkage, and sufficiently dense genomic sampling. Recent sequencing of Philippine ASFV strains from outbreaks in Luzon, the Visayas, and Mindanao confirmed continued circulation of closely related genotype II viruses and identified shared marker patterns among some 2023 isolates, but the number of sequenced outbreaks remains too small to reconstruct all inter-island transmission pathways [16]. The regional results should therefore be interpreted as evidence of asynchronous detected activity, not proof of a specific direction or route of spread.

Regional record counts and regional sample positivity also represent different dimensions of the surveillance burden. A region can accumulate many positive records under intensive testing while maintaining moderate positivity, whereas a smaller, highly targeted testing program can produce fewer records but a high proportion positive. Very low values may indicate limited detected activity, extensive low-risk screening, or insufficient surveillance. Accordingly, regional prioritization should combine record counts, tested-sample volume, positivity, onset and peak timing, and data completeness rather than ranking regions on a single indicator. A single well-supported introduction into a previously unaffected island population may be more consequential than numerous records generated by repeated sampling in an already affected area.

### 4.4. Late-Year Concentration

September and October had the highest mean national seasonality indices, but their 95% confidence intervals included the reference value of 1. The result therefore indicates a suggestive late-year concentration rather than a statistically established nationwide seasonal cycle. This distinction is important because only four complete years contributed to the analysis. The early epidemic phase exerted a strong influence on the series, and exploratory regional patterns were heterogeneous. The lower December index was more consistent across years, but the month-specific estimates should still be viewed as descriptive features of the surveillance series rather than direct evidence of seasonally varying transmission probability.

Several non-exclusive mechanisms could produce a late-year concentration. Commercial and behavioral mechanisms include increased pig and pork movement, greater market demand before Christmas and New Year, local festivities, and restocking or finishing cycles in backyard production systems. Movement through formal and informal value chains creates repeated opportunities for contaminated pigs, products, vehicles, and equipment to connect holdings [12,26]. Environmental conditions may also contribute. ASFV can persist on contaminated fomites, with survival generally longer at lower environmental temperatures and on some porous materials. This highlights the importance of cleaning and disinfection along the production and transport chain [27]. Wet conditions, mud, organic matter, and difficult-to-disinfect surfaces may increase the operational challenge even where ambient temperatures are relatively high.

An administrative explanation is also plausible. Pre-holiday movement requirements and shipment testing may increase the number of submissions during the same months in which market activity intensifies. Such an effect would elevate detected records without necessarily increasing the underlying proportion positive. Comparison of counts with monthly tested-sample volume and stream-specific positivity would be needed to separate this mechanism formally. The present annual positivity analysis partially addresses denominator effects, but the study did not model monthly trade volume, rainfall, flooding, or policy implementation intensity.

The Philippine pattern should not be assumed to mirror seasonality in temperate settings. In European data, domestic-pig outbreaks often peaked in summer, while wild-boar patterns varied across winter, spring, and summer depending on country and ecological context [28]. The present database comprised domestic-production records and contained no dedicated wild-suid surveillance stream. European wild-boar-driven mechanisms therefore provide a useful contrast but cannot be transferred directly to the Philippine results. Operationally, the late-year point estimates justify heightened vigilance during the third and early fourth quarters, but not a uniform national seasonal rule. Regional surveillance histories and local movement calendars should determine the timing and intensity of preventive action.

### 4.5. Interpretation of Joinpoint Regression and the EDR

Joinpoint regression and EDR contributed information at different temporal scales. The joinpoint model identified the broad structural signature of the epidemic: a sharp national increase from June to September 2020, a sharp decline through December 2020, and no clearly supported sustained linear trend during most of the later period. This model-based pattern reinforces the interpretation that 2020 was exceptional in national magnitude, whereas subsequent activity occurred around a lower and more variable level.

The exact breakpoint structure nevertheless requires caution. Residual diagnostics identified autocorrelation, non-normality, and non-constant variance. These are common concerns in veterinary surveillance time series and can affect conventional regression uncertainty [19]. In the AR(1) sensitivity analysis, the changes associated with the three 2020 breakpoints remained supported, whereas the August 2022 change in slope did not. The most defensible inference is therefore the robust mid-2020 rise and post-peak decline, not the claim that every selected month represents a precisely dated biological transition. The slopes describe changes in monthly positive-record counts and should not be interpreted as transmission rates.

The 14-day EDR revealed shorter expansion and contraction episodes within the broader joinpoint-defined phases. Twenty-five sustained expansions and 28 contractions were detected, including the largest and longest expansions during 2020 but additional episodes in 2021, 2022, and 2023. This demonstrates why the absence of a later positive long-term slope does not imply uninterrupted decline. Short regional or multi-regional pulses can occur within a broadly flat national segment. EDR is a direct ratio of recent to preceding detected counts and is valuable because it is transparent and reproducible [21]. In this study, however, it should not be equated with R0, Rt, a generation-specific reproduction number, or a per-capita transmission rate because the analysis lacked a defined population at risk, transmission chains, and generation-interval information.

The window sensitivity analysis provides an empirical basis for the 14-day specification. The 7-day series produced substantially larger extremes and weak agreement with the longer windows, consistent with sensitivity to weekly reporting cycles, investigation clusters, and small denominators. The 14- and 21-day series were more concordant, while the 14-day window retained greater responsiveness. Thus, 14 days represents an operational compromise between noise reduction and timely detection of changing momentum rather than a claim that all ASF transmission generations last exactly two weeks.

EDR performance was also scale dependent. It was calculable throughout the evaluated national series. However, undefined days were common in many regional series because the preceding window contained no positive records. Regions such as NCR and BARMM had undefined values for most evaluated days. Regional EDR estimates should therefore not be interpreted as equally stable or comparable. Before EDR is adopted as a subnational alert tool, authorities should report the proportion of valid days, consider longer windows or alternative indicators for sparse regions, and interpret EDR together with testing volume and surveillance purpose.

### 4.6. Competing Mechanisms and the Context of National Control Measures

Several mechanisms may have contributed to the post-2020 reduction in detected intensity. Mortality, emergency sale, depopulation, and temporary closure of pig holdings could have reduced both the susceptible population and the connectivity of affected production areas. The socioeconomic disruption associated with ASF control in Philippine smallholder systems was substantial and extended beyond direct animal losses [10]. Susceptible-host depletion is therefore a credible partial explanation for the rapid decline following the 2020 peak.

Host depletion alone, however, does not account for the full pattern. Repopulation and recovery programs progressively returned susceptible pigs to previously affected holdings, and the low positivity of the repopulation stream documents extensive testing of farms seeking to resume production. Continued detections and later peaks in previously less affected regions further indicate that susceptible populations remained available for new introduction and local amplification. The later epidemic pattern is therefore more plausibly explained by a combination of reduced national-scale connectivity, localized persistence, reintroduction, restocking, and changing surveillance than by complete epidemic burnout.

Behavioral adaptation and programmatic learning may also have contributed. Repeated exposure to ASF can change farmer risk perception, marketing decisions, and willingness to adopt biosecurity. Likewise, veterinary services may improve investigation, laboratory routing, zoning, and risk communication over time [25,29]. At the same time, fatigue, distrust, or fear of uncompensated depopulation may encourage emergency sale or non-reporting. Compensation policies can increase reporting when they reduce the expected loss from notification, but delayed or insufficient payment can have the opposite effect. These mechanisms could influence both the true epidemic and the probability that infection entered the national database.

The study period also overlapped the COVID-19 pandemic. Restrictions and disruptions during 2020–2021 may have reduced legal animal movement and some opportunities for ASF dissemination. However, they may also have delayed field investigation, sample transport, laboratory processing, and routine surveillance. Disruption of formal supply chains could additionally have promoted informal trade or emergency selling. Because these pathways operate in opposing directions, the pandemic cannot be invoked as a simple explanation for either the increase or decline in detected ASF activity.

The policy chronology shows that the national response expanded from emergency investigation, depopulation, compensation, zoning, and movement restrictions in 2019–2020 toward community-based surveillance, recovery, zone progression, standardized diagnostics, biosecurity classification, sentinel procedures, and movement certification in 2021–2023. These measures provide essential context for the changing surveillance system and may have influenced both transmission and detection. Nonetheless, temporal concurrence between an issuance and a joinpoint or EDR episode is not evidence of policy effect. Policies were frequently introduced in response to worsening epidemic conditions. Several measures overlapped. Implementation likely varied among local governments, and the analysis contained no counterfactual or quantitative implementation measure. The policy comparison should therefore remain descriptive.

The retrospective analyses are useful for identifying how surveillance signals changed and for determining when standing control activities might be intensified. They do not, by themselves, predict the next affected region or establish that measures are needed only during a particular epidemic phase. Surveillance, rapid investigation, biosecurity, movement management, and appropriate compensation remain necessary throughout expansion, contraction, and low-activity periods.

### 4.7. Viral Context, Study Strengths, Limitations, and Future Priorities

The viral context is important when interpreting the period studied. Genomic analysis of ten Philippine strains collected from outbreaks between 2021 and 2023 classified all as p72 genotype II and CD2v serogroup 8, with multiple markers closely related or identical to the Georgia 2007/1 lineage [16]. The present results can therefore be viewed as a national surveillance baseline for the genotype II-dominant phase. That baseline may not remain stable as the virus evolves. Highly virulent recombinant genotype I/II viruses have been detected in China and Vietnam, and experimental evidence indicates that some genotype II-derived live-attenuated vaccine immunity does not protect against these recombinants [30,31]. The surveillance database did not contain sufficiently dense genomic or vaccination metadata to evaluate variant-specific dynamics or vaccine effects. Historical trends should therefore not be extrapolated uncritically to future viral or vaccination contexts.

The study has several strengths. It used a national dataset spanning all administrative regions, explicitly separated laboratory-record counts from individual-sample positivity, stratified results by surveillance purpose, and combined national and regional descriptions with methods operating at monthly and daily scales. Exact-row deduplication prevented a large number of identical records from distorting the epidemic curves and derived indicators. The incomplete 2024 extension was separated from the primary analysis, avoiding an artificial decline caused by missing surveillance months. The convergence of record counts, tested-sample denominators, stream-specific positivity, regional trajectories, joinpoint regression, and EDR provides a more defensible interpretation than any single indicator could provide.

Several limitations remain. A laboratory record was one deduplicated database row rather than a unique infected pig, farm, household, premises, or epidemiologically linked outbreak, and without a unique premises identifier repeated submissions from the same holding could not be linked nor the number of affected pigs per event determined. Program submissions were not a probability sample and varied by surveillance purpose, region, diagnostic access, and time, so sample positivity describes tested samples only. Collection or detection dates lagged infection and onset by an unknown interval, and the analysis did not incorporate pig-population denominators, farm density, movement networks, climatic variables, genomic linkage, or measures of policy implementation. The seasonality analysis rested on only four complete years and could not separate climatic, commercial, behavioral, and administrative mechanisms. The ordinary least-squares joinpoint model retained residual autocorrelation and distributional departures, making the exact later breakpoint less secure than the broad 2020 structural change, and the EDR was sensitive to window length and frequently undefined in sparse regional series. Finally, the dataset represented domestic-pig surveillance with no dedicated wild-suid stream, so no inference can be made about the contribution of Philippine wild pigs to the observed domestic-sector dynamics.

Fifth, the seasonality analysis was based on only four complete years and cannot distinguish climatic, commercial, behavioral, and administrative mechanisms. Sixth, the ordinary least-squares joinpoint model retained residual autocorrelation and distributional departures, making the exact later breakpoint less secure than the broad 2020 structural change. Seventh, EDR was sensitive to window length and frequently undefined in sparse regional series. Finally, the dataset represented domestic-pig surveillance and contained no dedicated wild-suid stream. No inference can therefore be made about the contribution of Philippine wild pigs to the observed domestic-sector dynamics.

Future surveillance would benefit from stable unique premises and specimen identifiers, standardized collection, onset, reporting, and confirmation dates, and complete metadata on surveillance purpose and diagnostic method. Linking laboratory records with pig-population denominators, legal and informal movement networks, genomic sequences, weather and flooding data, depopulation and repopulation records, compensation timing, and vaccination status would allow stronger discrimination among surveillance effects, local persistence, and repeated introduction. Operational dashboards should retain the multi-indicator principle used here. Positive-record counts should be interpreted with testing volume and stream-specific positivity. National signals should be decomposed regionally; and EDR should be accompanied by valid-day completeness. This framework is particularly relevant to geographically fragmented and smallholder-dominated production systems, where a lower national signal can coexist with substantial local risk.

## 5. Conclusions

National surveillance data indicate that detected ASF activity in the Philippines was most intense during 2020, when the monthly positive-record maximum, the highest annual sample positivity, and the strongest joinpoint-defined changes occurred. Thereafter, sample positivity declined while testing volume expanded substantially. This provides stronger evidence of a reduction in the proportion of positive samples among tested animals than would a decline in raw record counts alone, but it does not establish a corresponding decline in national incidence or prevalence. The national pattern also showed marked regional asynchrony. Several Luzon regions peaked early, whereas later peaks occurred in parts of Mindanao, and the Visayas. September and October showed the highest seasonal point estimates, but the uncertainty intervals did not support a fixed nationwide seasonal cycle.

Joinpoint regression and EDR provided complementary perspectives. The joinpoint analysis identified a robust national rise and decline during 2020. Meanwhile, the 14-day EDR showed that shorter expansion and contraction episodes continued through 2023. The later epidemic period was therefore characterized by lower national positivity without disappearance of regional risk. Surveillance and control should remain continuous across epidemic phases, while regional investigation, movement management, biosecurity support, and laboratory capacity should be adapted to local trajectories and surveillance-stream objectives. Future analyses would be strengthened by unique premises identifiers, pig-population denominators, standardized onset and reporting dates, movement-network data, genomic linkage, and measures of policy implementation. Because the present analyses were observational and descriptive, temporal correspondence with control measures should not be interpreted as proof of effectiveness, and the identified historical patterns should not be treated as deterministic forecasts of future ASF activity.

## Figures and Tables

**Table 1:** Surveillance composition and ASF sample positivity by principal surveillance stream, Philippines, August 2019–December 2023.

Surveillance Stream	Laboratory Records	Positive Records	Tested Samples	Positive Samples	Positivity (%)
Disease Investigation	8854	6512	27,725	17,512	63.2
Surveillance	31,846	2192	117,014	5044	4.3
Local Shipment	4846	179	149,941	1674	1.1
Repopulation	5304	46	29,952	189	0.6

Sample positivity was calculated as positive samples divided by positive plus negative samples. The four streams differed in purpose and pre-test probability and were therefore interpreted separately.

**Table 2:** Annual ASF laboratory-record, tested-sample, and positivity indicators, Philippines, 2019–2023.

Year	Laboratory Records	Positive Records	Tested Samples	Positive Samples	Overall Positivity (%)	Routine Positivity (%)
2019	2299	544	35,569	3048	8.6	4.9
2020	4678	2846	41,035	8448	20.6	17.9
2021	12,164	2286	68,682	5330	7.8	7.2
2022	14,272	1513	77,122	3566	4.6	3.0
2023	17,439	1740	102,333	4027	3.9	3.3

The 2019 values represent a partial observation period beginning on 8 August 2019.

**Table 3:** Segment-specific slopes from the BIC-selected joinpoint regression of monthly ASF-positive laboratory records.

Segment	Start	End	Slope *	95% Confidence Interval		Direction
1	Aug 2019	Jun 2020	−0.99	−17.19	15.21	No clear linear change
2	Jun 2020	Sep 2020	144.40	88.40	200.41	Increasing
3	Sep 2020	Dec 2020	−129.31	−179.35	−79.28	Decreasing
4	Dec 2020	Aug 2022	−2.36	−7.64	2.92	No clear linear change
5	Aug 2022	Dec 2023	2.83	−4.19	9.85	No clear linear change

* Slope (records/month). Confidence intervals are conditional on the selected month-boundary breakpoint locations.

**Table 4:** National EDR window-length sensitivity analysis.

Window	Valid EDR Days	Median	Mean	Maximum	Expansion Episodes	Contraction Episodes
7 days	1591	1.00	1.21	18.50	5	6
14 days	1580	0.98	1.17	7.80	25	28
21 days	1566	1.00	1.17	4.73	19	22

The 7-day specification had three evaluable days with undefined EDR because the preceding window total was zero; the 14- and 21-day national specifications had none.

**Table 5:** Major sustained 14-day EDR expansion episodes, ranked by positive laboratory records.

Start	End	Duration (Days)	Median EDR	Peak EDR	Positive Records
13 Aug 2020	14 Sep 2020	33	1.34	3.53	755
08 Jan 2020	17 Feb 2020	41	1.87	7.50	403
05 Jun 2020	11 Aug 2020	68	1.44	2.15	365
06 Sep 2021	28 Sep 2021	23	2.12	4.42	282
08 Jan 2021	04 Feb 2021	28	1.72	3.10	253
05 Oct 2022	30 Oct 2022	26	1.17	1.60	253
23 May 2023	23 Jun 2023	32	1.60	2.87	220
15 Jun 2021	15 Jul 2021	31	1.41	2.41	207
29 Aug 2022	22 Sep 2022	25	1.54	2.53	174
11 Sep 2023	01 Oct 2023	21	1.60	1.95	156

## Data Availability

Access to the data is subject to approval by the National African Swine Fever Prevention and Control Program (NASFPCP). Researchers seeking access to the data must submit an official request letter to the NASFPCP.

## Supplementary Materials

The following supporting information can be downloaded at: https://www.mdpi.com/article/10.3390/ani16172811/s1, Figure S1: Regional ASF-positive laboratory-record curves with a common *y*-axis, Philippines, 2019–2023; Figure S2: Regional ASF-positive laboratory-record curves with region-specific *y*-axes, Philippines, 2019–2023; Figure S3: Descriptive ASF sample positivity by administrative region; Figure S4: Exploratory regional monthly seasonality indices. Cells are displayed when at least two region-years contributed. Sparse and late-emergent regional series should not be interpreted as stable regional seasonality; Figure S5: ASF sample positivity by surveillance stream; Figure S6: Normal Q–Q plot of residuals from the selected joinpoint model; Figure S7: Residuals versus fitted values for the selected joinpoint model; Figure S8: Sensitivity of the Estimated Dissemination Ratio to 7-, 14-, and 21-day windows. Free *y*-axes emphasize the greater volatility of the 7-day specification; Figure S9: Incomplete 2024 surveillance extension. The 2024 records are descriptive only and were excluded from primary temporal models because coverage was concentrated in January with one December record; Figure S10: Temporal correspondence of monthly ASF-positive laboratory records and major national control measures. The overlay is descriptive; temporal coincidence does not establish policy effectiveness. Table S1: Data-quality rules and analytic consequences; Table S2: Prespecified analysis parameters; Table S3: National ASF measures included in the descriptive temporal overlay; Table S4: Analytic flow from the merged surveillance file to the primary 2019–2023 dataset; Table S5: First recorded positive month, peak month, and cumulative positive laboratory records by administrative region; Table S6: Regional ASF sample positivity and completeness of the 14-day EDR series; Table S7: Mean monthly seasonality indices for ASF-positive laboratory records, 2020–2023; Table S8: (A) Candidate joinpoint models and BIC model selection. (B) Joinpoint residual diagnostic and AR(1) sensitivity coefficients; Table S9: Pairwise agreement among 7-, 14-, and 21-day EDR specifications; Table S10: All sustained national 14-day EDR expansion and contraction episodes; Table S11: (A) Summary of the incomplete 2024 surveillance extension. (B) Monthly distribution of the incomplete 2024 extension; Table S12: Descriptive ASF indicators by recorded farm type.

## Abbreviations

The following abbreviations are used in this manuscript:

ASF	African swine fever
EDR	Estimated Dissemination Ratio
BAI	Bureau of Animal Industry
NASFPCP	National African Swine Fever Prevention and Control Programme
