# Supplementary material for: Quantifying ASF Outbreak Dynamics in the Philippines (2019–2023) Using Joinpoint Regression and the Estimated Dissemination Ratio
Source: Animals (Basel). 2026 Sep 7;16(17):2811. doi: 10.3390/ani16172811 (PMC13564805; doi:10.3390/ani16172811)
Supplement: Supplementary file 1 [file animals-16-02811-s001.zip › animals-4418960-supplementary.pdf]

Supplementary Materials

Quantifying ASF Outbreak Dynamics in the Philippines (2019–2023) Using Joinpoint Regression and the Estimated Dissemination Ratio

Supplementary Methods, Tables, and Figures

Contents

- Supplementary Methods and Reproducibility Details
- Supplementary Tables S1–S12
- Supplementary Figures S1–S10

Supplementary Methods and Reproducibility Details

S1. Detailed Analytic Flow

The source file contained 102,808 rows. Exact comparison across all 19 original fields identified 14,853 duplicate-valued groups and 51,638 additional identical rows. One row per group was retained, leaving 51,170 rows. Twenty-two rows with invalid qualitative result labels were excluded, producing 51,148 valid deduplicated records. Of these, 50,852 belonged to the primary 2019-2023 period and 296 to the incomplete 2024 extension.

Supplementary Table S1. Data-quality rules and analytic consequences.

Issue	Operational definition	Action
Exact duplicate	All 19 original fields identical	Retain one row; remove additional identical rows
Invalid qualitative result	Result not Positive, Negative, or Not Tested	Exclude from all analyses
Missing negative count	Recoverable from total - positive - untested	Reconstruct when arithmetic is unambiguous
Total/component imbalance	Total differs from positive + negative + untested	Flag; use positive + negative for positivity
Denominator ineligible	Positive + negative absent or nonpositive	Exclude only from sample-positivity denominator
Incomplete 2024 coverage	Records in January and one December record only	Descriptive extension; exclude from primary temporal models

S2. Standardization Rules

Administrative-region labels were standardized to NCR, CAR, Regions I-XIII, IV-A, IV-B, and BARMM. Surveillance-purpose labels were standardized using text rules that mapped disease-investigation, repopulation, shipment or movement, and surveillance variants to the four principal streams. Original values were retained in audit outputs. Coordinates were treated as valid when longitude was between 115 and 130 degrees east and latitude between 3 and 23 degrees north.

S3. Seasonality Calculations

The year-standardized seasonality index controls for between-year differences in the overall monthly level by scaling each month to the mean of the 12 months in the same year. For each calendar month, the reported estimate was the arithmetic mean of the four annual indices from 2020-2023. The confidence interval was:

$$\bar{SI}_m \pm t_{0.975,3} \times s_m / \sqrt{4} \quad \text{(S1)}$$

In Equation (S1), $\bar{SI}_m$ is the mean seasonality index for calendar month m across the four complete years; $t_{0.975,3}$ is the 97.5th percentile of the t distribution with three degrees of freedom; s_m is the between-year standard deviation of the four month-specific indices; and $\sqrt{4}$ is the square root of the number of contributing years.

Because only four complete years were available, the intervals were expected to be wide. No multiple-comparison-adjusted significance claims were made across the 12 months.

S4. Joinpoint Candidate Search and BIC

For each candidate number of joinpoints $k = 0,...,5$, segmented regression was used to obtain continuous starting values. A local integer search considered nearby calendar-month boundaries while enforcing the minimum three-month segment length. For each fixed breakpoint set, a linear hinge model was fitted by ordinary least squares.

$$\mathrm{BIC} = n \ln(\mathrm{RSS} / n) + p \ln(n) \tag{S2}$$

In Equation (S2), BIC is the Bayesian Information Criterion; n is the number of monthly observations; RSS is the residual sum of squares from the candidate model; and p is the number of estimated model parameters, including the ordinary regression coefficients and one parameter for each breakpoint location.

Here n was the number of monthly observations and p included the ordinary regression coefficients plus one parameter for each estimated breakpoint location. Conditional segment-slope confidence intervals were derived from the fitted coefficient covariance matrix. Because breakpoint-selection uncertainty was not propagated into these intervals, the exact breakpoint dates and slope intervals were interpreted cautiously.

S5. EDR Episode Algorithm

For each day and window length, the numerator was the sum of positive records during the most recent w days and the denominator was the sum during the immediately preceding w days. The first 2w - 1 days were not evaluable. A zero denominator produced an undefined EDR. Runs were formed from consecutive daily states: Expansion for EDR > 1, Contraction for EDR < 1, Stable for EDR = 1, and Undefined when an estimate could not be calculated. Only expansion or contraction runs lasting at least 14 days were retained as sustained episodes.

$$\mathrm{EDR}_t(w) = \left[\Sigma_{i=t-w+1}^{t} x_i\right] / \left[\Sigma_{i=t-2w+1}^{t-w} x_i\right] \tag{S3}$$

In Equation (S3), $\mathrm{EDR}_t(w)$ is the Estimated Dissemination Ratio on day t for a window of w days; x_i is the number of positive laboratory records on day i; the numerator is the total during the most recent w days; and the denominator is the total during the immediately preceding, non-overlapping w-day period. The ratio was undefined when the denominator was zero.

S6. Policy-Timeline Analysis

Policy dates were drawn from official issuances. The overlay was designed to show temporal co-occurrence only. Policies were not coded as binary interventions in the joinpoint or EDR models, and no lag, level change, slope change, or counterfactual effect was estimated.

S7. Reproducibility Parameters

Supplementary Table S2. Prespecified analysis parameters.

Parameter	Value
Input file	merge 2019-2024 clean.csv
Raw imported rows	102808
Exact duplicate rows removed	51638
Rows with invalid result labels excluded	22
Primary study start	2019-08-08
Primary study end	2023-12-31
Incomplete extension start	2024-01-01
Incomplete extension end	2024-12-31
Incomplete extension records	296
Temporal analytic unit	One count per deduplicated qualitative positive laboratory record
Positivity denominator	positive_n / (positive_n + negative_n_clean)
Complete seasonality years	2020, 2021, 2022, 2023
Maximum joinpoints	5
Minimum segment length, months	3
Primary EDR window, days	14
Sustained episode minimum, days	14
Random seed	20260714

S8. Policy Timeline Used for the Descriptive Overlay

Supplementary Table S3. National ASF measures included in the descriptive temporal overlay.

Issue date	Measure	Official issuance	Analytic role	Main actions
2019-08-18	Emergency investigation and depopulation guidance	DA Administrative Order 31, Series of 2019	Descriptive temporal correspondence only	Mandatory reporting and investigation of suspected ASF; culling of infected and in-contact pigs; carcass disposal and premises disinfection
2019-09-09	1-7-10 protocol	DA Administrative Order 7, Series of 2019	Descriptive temporal correspondence only	Zoning by radius around a confirmed case: depopulation within 1 km, active surveillance within 7 km, and monitoring and movement control within 10 km
2019-12-10	National Zoning and Movement Plan	DA Administrative Circular 12, Series of 2019	Descriptive temporal correspondence only	Establishes color-coded zoning (infected, buffer, surveillance, and free) with corresponding pig- and pork-movement rules between zones
2020-02-05	Strict national and local implementation of zoning	Office of the President Administrative Order 22	Descriptive temporal correspondence only	Directs all national agencies and local government units to enforce the national zoning and movement plan
2020-02-21	National Task Force on Animal-Borne Diseases	Office of the President Executive Order 105	Descriptive temporal correspondence only	Creates a national inter-agency task force to coordinate the animal-borne-disease response

Issue date	Measure	Official issuance	Analytic role	Main actions
2020-05-29	Revised swine depopulation guidance	DA Administrative Order 22, Series of 2020	Descriptive temporal correspondence only	Updates depopulation procedures, culling radius, and indemnification of affected raisers
2020-12-22	Intensified ASF disease-control program	DA Administrative Order 43, Series of 2020	Descriptive temporal correspondence only	Integrated national program combining intensified surveillance, biosecurity, zoning, and repopulation activities
2021-02-08	Bantay ASF sa Barangay	DA Administrative Order 8, Series of 2021	Descriptive temporal correspondence only	Community-based (barangay-level) early detection and reporting, engaging local officials and raisers
2021-11-26	Recovery, zone progression, and declaration of freedom	DA Administrative Order 30, Series of 2021	Descriptive temporal correspondence only	Criteria for sentinel-based repopulation, progression of zones toward lower risk, and declaration of ASF freedom
2022-03-21	Local implementation of Bantay ASF sa Barangay	DA-DILG Joint Memorandum Order 1, Series of 2022	Descriptive temporal correspondence only	Directs local government units to operationalize barangay-level surveillance and reporting
2022-06-13	Technical standards for biosecurity, diagnostics, sentinel procedures, and sampling	BAI Memorandum Circulars 20, 21, 22, and 26, Series of 2022	Descriptive temporal correspondence only	Standardized farm-biosecurity classification, laboratory-diagnostic, sentinel, and sampling protocols
2022-08-04	ASF-free movement certification requirements	BAI Memorandum Circular 35, Series of 2022	Descriptive temporal correspondence only	Certification requirements for moving pigs and pork products originating from ASF-free areas

Issue date	Measure	Official issuance	Analytic role	Main actions
2023-07-14	Movement of pork products from Pink to Yellow Zones	DA Administrative Circular 6, Series of 2023	Descriptive temporal correspondence only	Conditional movement of processed pork products from buffer (pink) to surveillance (yellow) zones under specified requirements
2023-08-14	Public-private movement audit	BAI Memorandum Circular 37, Series of 2023	Descriptive temporal correspondence only	Joint public–private audit of pig- and pork-movement compliance

Under the National Zoning and Movement Plan, areas were classified by ASF status: infected (red) zones, where ASF was confirmed and depopulation applied; buffer (pink) zones immediately surrounding infected zones, with heightened surveillance and movement control; surveillance (yellow) zones under active monitoring; and free or protected (green) zones without detected ASF. Movement of pigs and pork products was regulated according to zone. Action descriptions summarize the principal operational content of each issuance; readers are referred to the primary documents for the full provisions.

Supplementary Tables

Supplementary Table S4. Analytic flow from the merged surveillance file to the primary 2019–2023 dataset.

Analytic stage	Number of records
Rows in imported merged CSV	102,808
Exact duplicate-valued groups	14,853
Extra exact duplicate-valued rows removed	51,638
Rows after exact-row deduplication	51,170
Rows with missing or invalid date	0
Rows with invalid qualitative result label	22
Valid exact-row-deduplicated records	51,148
Valid records in incomplete 2024 extension	296
Records in primary analytic period	50,852
Positive laboratory records in primary period	8,929
Records eligible for sample-positivity denominator	50,783
Records with valid region	50,852
Records with valid geographic coordinates	50,852
Primary-period representatives of duplicate-valued groups	14,787
Rows with total/component count imbalance	350
Rows with reconstructed negative count	2
Qualitative result/count disagreements	0

Supplementary Table S5. First recorded positive month, peak month, and cumulative positive laboratory records by administrative region.

Region	Administrative name	First positive	Last positive	Peak month	Peak count	Total positive
III	Central Luzon	Aug 2019	Dec 2023	Feb 2020	193	1,156
IV-A	CALABARZON	Aug 2019	Dec 2023	Aug 2020	136	819
CAR	Cordillera Administrative Region	Sep 2019	Dec 2023	May 2021	33	301
I	Ilocos Region	Sep 2019	Nov 2023	Sep 2021	260	1,067
NCR	National Capital Region	Sep 2019	Jun 2020	Sep 2019	11	26
II	Cagayan Valley	Oct 2019	Dec 2023	Sep 2020	419	1,188
IV-B	MIMAROPA	Oct 2019	Dec 2023	Nov 2023	104	394
XI	Davao Region	Jan 2020	Mar 2023	Jan 2021	87	521
V	Bicol Region	Feb 2020	Dec 2023	Jan 2021	74	526
XII	SOCCSKSARGEN	Feb 2020	Dec 2023	Sep 2022	119	705
X	Northern Mindanao	Nov 2020	Dec 2023	Apr 2021	76	373
XIII	Caraga	Nov 2020	Dec 2023	Jul 2021	45	490
VIII	Eastern Visayas	Jan 2021	Dec 2023	Mar 2022	59	437
IX	Zamboanga Peninsula	May 2022	Aug 2023	Jul 2022	40	133
VI	Western Visayas	Oct 2022	Oct 2023	Jun 2023	151	545
VII	Central Visayas	Nov 2022	Dec 2023	Aug 2023	60	246
BARMM	Bangsamoro Autonomous Region in Muslim Mindanao	Mar 2023	Mar 2023	Mar 2023	2	2

Supplementary Table S6. Regional ASF sample positivity and completeness of the 14-day EDR series.

Region	Records	Positive records	Tested samples	Positive samples	Positivity (%)	Undefined EDR days (%)
BARMM	249	2	657	4	0.6	99.1
CAR	1,417	301	3,585	587	16.4	37.1
I	3,810	1,067	24,446	3,977	16.3	41.3
II	3,693	1,188	9,730	2,648	27.2	27.0
III	4,779	1,156	81,863	5,664	6.9	34.9
IV-A	11,454	819	71,183	2,485	3.5	18.4
IV-B	2,646	394	18,490	980	5.3	74.5
IX	1,177	133	4,674	327	7.0	85.1
NCR	121	26	863	128	14.8	91.8
V	4,350	526	18,960	1,119	5.9	43.2
VI	1,684	545	10,969	1,104	10.1	77.0
VII	1,274	246	5,658	476	8.4	79.4
VIII	1,983	437	3,808	683	17.9	53.2
X	1,618	373	17,196	755	4.4	59.1
XI	1,857	521	8,460	945	11.2	50.3
XII	6,300	705	37,112	1,436	3.9	47.6
XIII	2,440	490	7,087	1,101	15.5	42.3

Note: Undefined EDR days were evaluated days on which the preceding 14-day regional total was zero; the initial 27 days needed to form two complete windows were excluded from the denominator.

Supplementary Table S7. Mean monthly seasonality indices for ASF-positive laboratory records, 2020–2023.

Month	Mean index	SD	Lower 95% CI	Upper 95% CI	Descriptive p
January	0.96	0.30	0.48	1.44	0.823
February	1.06	0.36	0.49	1.64	0.757
March	0.75	0.21	0.42	1.09	0.102
April	0.56	0.32	0.06	1.07	0.070
May	0.58	0.36	0.01	1.15	0.102
June	0.80	0.44	0.10	1.51	0.436
July	0.93	0.12	0.74	1.13	0.356
August	1.11	0.61	0.14	2.08	0.739
September	1.98	0.78	0.75	3.21	0.086
October	1.58	0.49	0.80	2.37	0.098
November	1.08	0.35	0.53	1.64	0.669
December	0.58	0.15	0.34	0.83	0.012

Note: P values are unadjusted one-sample t tests against an index of 1 and are descriptive. Only four complete years contributed.

Supplementary Table S8A. Candidate joinpoint models and BIC model selection.

Joinpoints	Breakpoints	RSS	Parameters	BIC	Selected
0	None	702987.2	2	511.06	No
1	2020-08-01	634688.4	4	513.58	No
2	2020-09-01; 2022-04-01	569075.4	6	515.74	No
3	2020-05-01; 2020-08-01; 2022-12-01	544365.8	8	521.33	No
4	2020-06-01; 2020-09-01; 2020-12-01; 2022-08-01	368745.1	10	508.62	Yes
5	2020-09-01; 2020-12-01; 2021-10-01; 2022-01-01; 2023-04-01	478241.7	12	530.35	No

Supplementary Table S8B. Joinpoint residual diagnostic and AR(1) sensitivity coefficients.

Analysis / term	Result	Interpretation
Ljung–Box residual test	Q = 17.63; df = 7; p = 0.014	Residual autocorrelation remained
AR(1) GLS: (Intercept)	Estimate = 125.76; 95% CI -15.24 to 266.75; p = 0.087	Sensitivity model
AR(1) GLS: time	Estimate = -0.56; 95% CI -20.59 to 19.47; p = 0.957	Sensitivity model
AR(1) GLS: hinge_1	Estimate = 160.65; 95% CI 81.56 to 239.74; p = 0.000	Sensitivity model
AR(1) GLS: hinge_2	Estimate = -305.72; 95% CI -422.76 to -188.67; p = 0.000	Sensitivity model
AR(1) GLS: hinge_3	Estimate = 143.90; 95% CI 79.71 to 208.10; p = 0.000	Sensitivity model
AR(1) GLS: hinge_4	Estimate = 3.96; 95% CI -10.53 to 18.44; p = 0.595	Sensitivity model

Supplementary Table S9. Pairwise agreement among 7-, 14-, and 21-day EDR specifications.

Window comparison	Paired valid days	Pearson r	Spearman ρ	Directional agreement (%)
7 vs 14	1,578	0.079	0.187	57.5
7 vs 21	1,566	0.012	0.087	53.0
14 vs 21	1,566	0.523	0.605	73.1

Supplementary Table S10. All sustained national 14-day EDR expansion and contraction episodes.

State	Start	End	Duration	Median EDR	Extreme EDR	Positive records
Expansion	11 Sep 2019	07 Oct 2019	27	2.38	7.80	95
Expansion	14 Oct 2019	31 Oct 2019	18	1.66	2.15	136
Contraction	01 Nov 2019	14 Nov 2019	14	0.68	0.65	84
Contraction	28 Nov 2019	17 Dec 2019	20	0.79	0.59	72
Contraction	19 Dec 2019	07 Jan 2020	20	0.59	0.16	33
Expansion	08 Jan 2020	17 Feb 2020	41	1.87	7.50	403
Contraction	04 Mar 2020	27 Apr 2020	55	0.55	0.26	150
Expansion	30 Apr 2020	13 May 2020	14	1.58	2.40	24
Expansion	05 Jun 2020	11 Aug 2020	68	1.44	2.15	365
Expansion	13 Aug 2020	14 Sep 2020	33	1.34	3.53	755
Contraction	25 Sep 2020	01 Nov 2020	38	0.59	0.24	319
Expansion	17 Nov 2020	02 Dec 2020	16	1.79	2.29	118
Contraction	03 Dec 2020	21 Dec 2020	19	0.64	0.41	78
Contraction	25 Dec 2020	07 Jan 2021	14	0.72	0.55	62
Expansion	08 Jan 2021	04 Feb 2021	28	1.72	3.10	253
Contraction	05 Mar 2021	22 Mar 2021	18	0.78	0.51	103
Contraction	01 Apr 2021	14 Apr 2021	14	0.70	0.48	79
Expansion	15 Apr 2021	28 Apr 2021	14	2.28	2.83	83
Contraction	29 Apr 2021	19 May 2021	21	0.51	0.34	68
Expansion	15 Jun 2021	15 Jul 2021	31	1.41	2.41	207
Contraction	16 Jul 2021	02 Aug 2021	18	0.73	0.49	78
Contraction	16 Aug 2021	01 Sep 2021	17	0.77	0.53	64
Expansion	06 Sep 2021	28 Sep 2021	23	2.12	4.42	282
Contraction	29 Sep 2021	12 Oct 2021	14	0.73	0.60	140

State	Start	End	Duration	Median EDR	Extreme EDR	Positive records
Contraction	09 Nov 2021	16 Dec 2021	38	0.74	0.51	191
Contraction	22 Dec 2021	09 Jan 2022	19	0.67	0.30	47
Expansion	10 Jan 2022	26 Jan 2022	17	1.46	3.05	74
Expansion	09 Feb 2022	22 Feb 2022	14	1.32	1.72	59
Contraction	23 Feb 2022	08 Mar 2022	14	0.47	0.41	43
Contraction	09 Apr 2022	26 Apr 2022	18	0.61	0.38	38
Expansion	27 Apr 2022	19 May 2022	23	1.73	2.73	119
Contraction	25 May 2022	21 Jun 2022	28	0.65	0.44	65
Expansion	22 Jun 2022	05 Jul 2022	14	1.95	2.24	65
Contraction	06 Jul 2022	19 Jul 2022	14	0.83	0.66	43
Expansion	29 Aug 2022	22 Sep 2022	25	1.54	2.53	174
Expansion	05 Oct 2022	30 Oct 2022	26	1.17	1.60	253
Contraction	31 Oct 2022	21 Nov 2022	22	0.47	0.21	85
Contraction	02 Dec 2022	26 Dec 2022	25	0.42	0.25	35
Expansion	31 Dec 2022	19 Jan 2023	20	1.62	2.58	58
Contraction	20 Jan 2023	02 Feb 2023	14	0.84	0.54	37
Expansion	08 Feb 2023	23 Feb 2023	16	2.14	3.00	89
Contraction	24 Feb 2023	12 Mar 2023	17	0.60	0.39	45
Contraction	24 Mar 2023	16 Apr 2023	24	0.69	0.46	30
Expansion	18 Apr 2023	10 May 2023	23	1.60	3.31	62
Expansion	23 May 2023	23 Jun 2023	32	1.60	2.87	220
Contraction	27 Jun 2023	16 Jul 2023	20	0.54	0.36	79
Expansion	17 Jul 2023	10 Aug 2023	25	1.26	2.00	147
Contraction	11 Aug 2023	10 Sep 2023	31	0.83	0.59	127
Expansion	11 Sep 2023	01 Oct 2023	21	1.60	1.95	156

State	Start	End	Duration	Median EDR	Extreme EDR	Positive records
Expansion	12 Oct 2023	26 Oct 2023	15	1.69	2.11	151
Contraction	27 Oct 2023	12 Nov 2023	17	0.64	0.45	106
Expansion	13 Nov 2023	26 Nov 2023	14	1.29	1.73	106
Contraction	27 Nov 2023	20 Dec 2023	24	0.75	0.48	112

Supplementary Table S11A. Summary of the incomplete 2024 surveillance extension.

Indicator	Value
Records	296
First recorded date	01 Jan 2024
Last recorded date	11 Dec 2024
Represented months	2
Positive laboratory records	83
Tested samples	1,942
Positive samples	136
Sample positivity (%)	7.0

Note: Incomplete extension only; excluded from primary temporal models because coverage was concentrated in January with one December record.

Supplementary Table S11B. Monthly distribution of the incomplete 2024 extension.

Month	Laboratory records	Positive records	Tested samples	Positive samples	Positivity (%)
Jan 2024	295	82	1941	135	7.0
Feb 2024	0	0	0	0	—
Mar 2024	0	0	0	0	—
Apr 2024	0	0	0	0	—
May 2024	0	0	0	0	—
Jun 2024	0	0	0	0	—
Jul 2024	0	0	0	0	—
Aug 2024	0	0	0	0	—
Sep 2024	0	0	0	0	—
Oct 2024	0	0	0	0	—
Nov 2024	0	0	0	0	—
Dec 2024	1	1	1	1	100.0

Supplementary Table S12. Descriptive ASF indicators by recorded farm type.

Farm type	Laboratory records	Positive records	Tested samples	Positive samples	Positivity (%)
Backyard	43,493	8,177	118,855	17,969	15.1
Commercial	5,161	328	181,507	4,609	2.5
Slaughterhouse	1,065	118	4,878	373	7.6
Semi-Commercial	822	260	11,283	1,107	9.8
Stockyard	194	26	5,670	184	3.2
Breeder	67	7	1,783	88	4.9
Others	33	12	571	86	15.1
Meat Shop	13	1	143	3	2.1
Holding Yard	3	0	50	0	0.0
Meatshop	1	0	1	0	0.0

Supplementary Figures

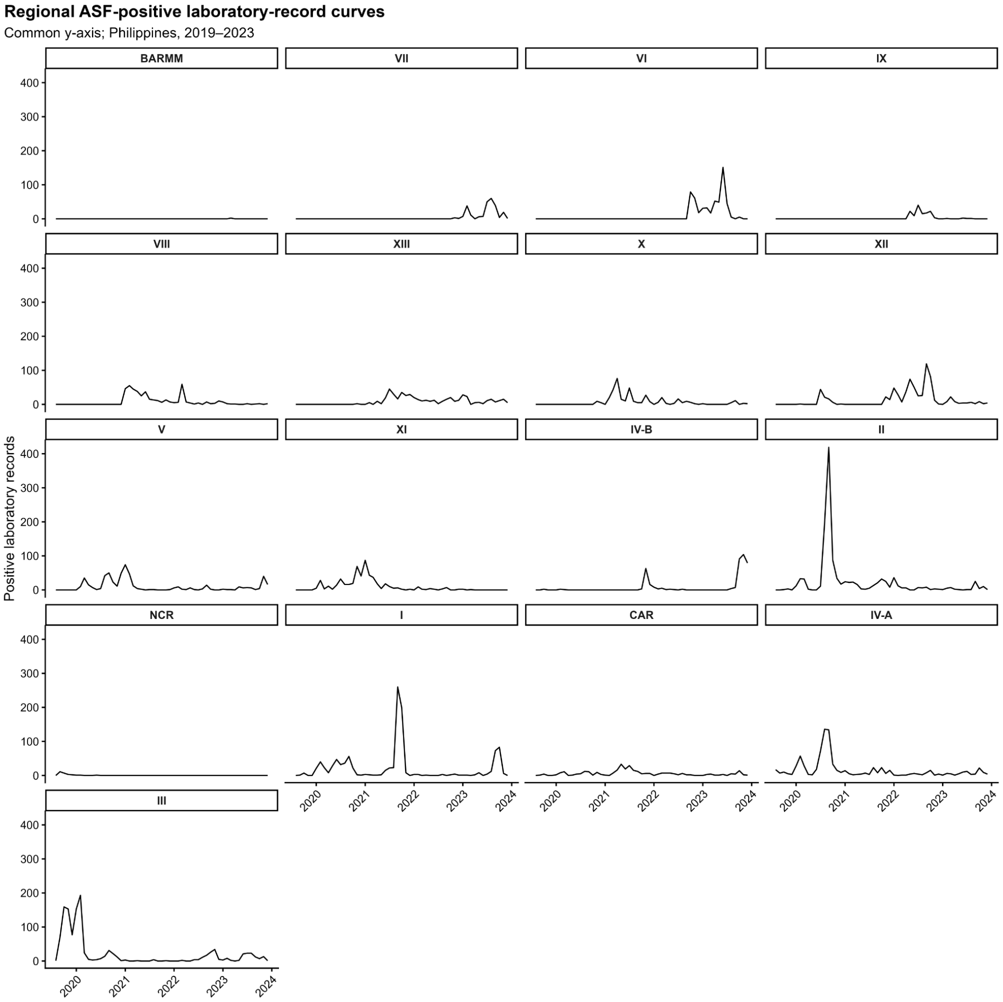

Supplementary Figure S1. Regional ASF-positive laboratory-record curves with a common y-axis, Philippines, 2019–2023.

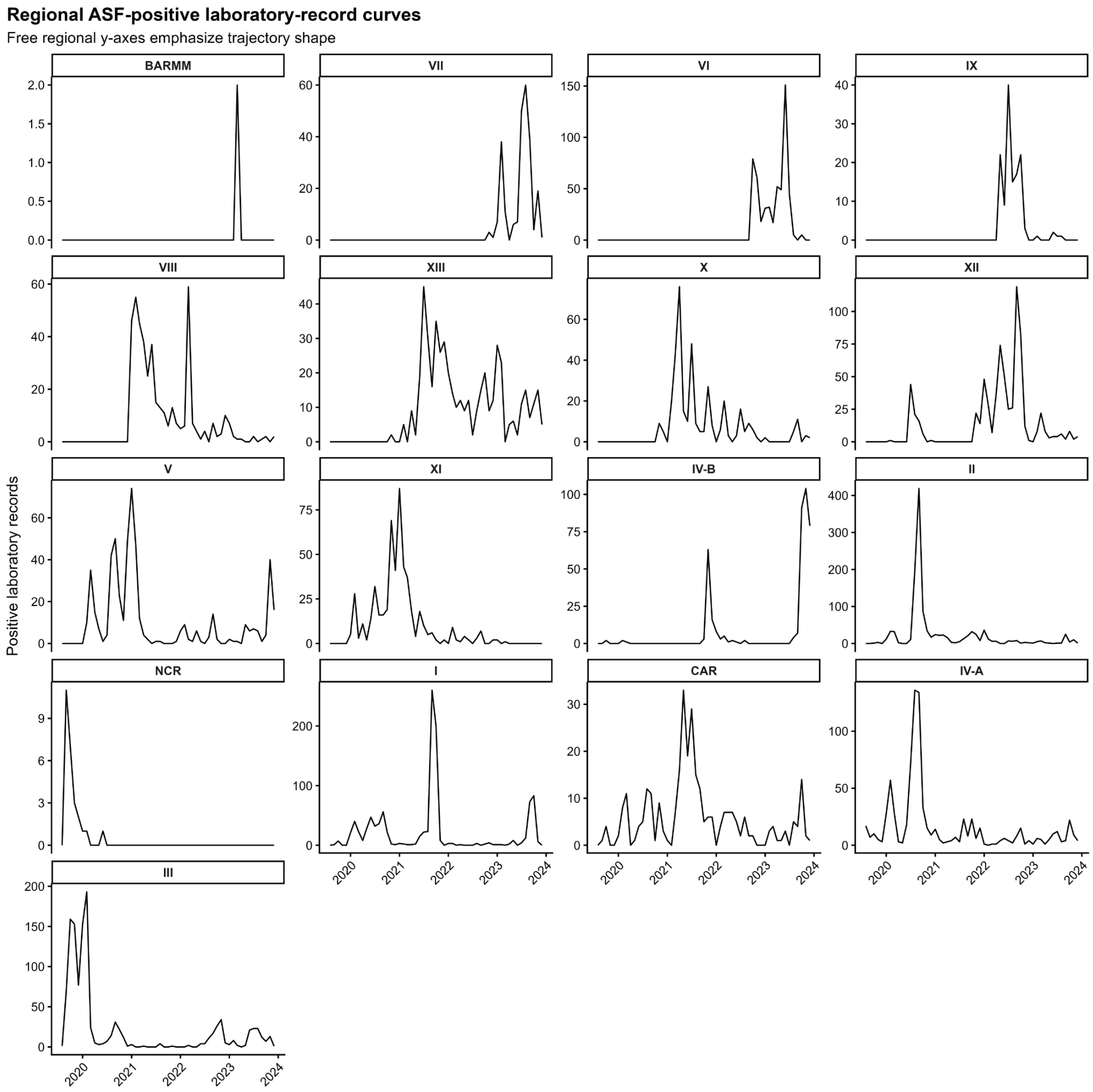

Supplementary Figure S2. Regional ASF-positive laboratory-record curves with region-specific y-axes, Philippines, 2019–2023.

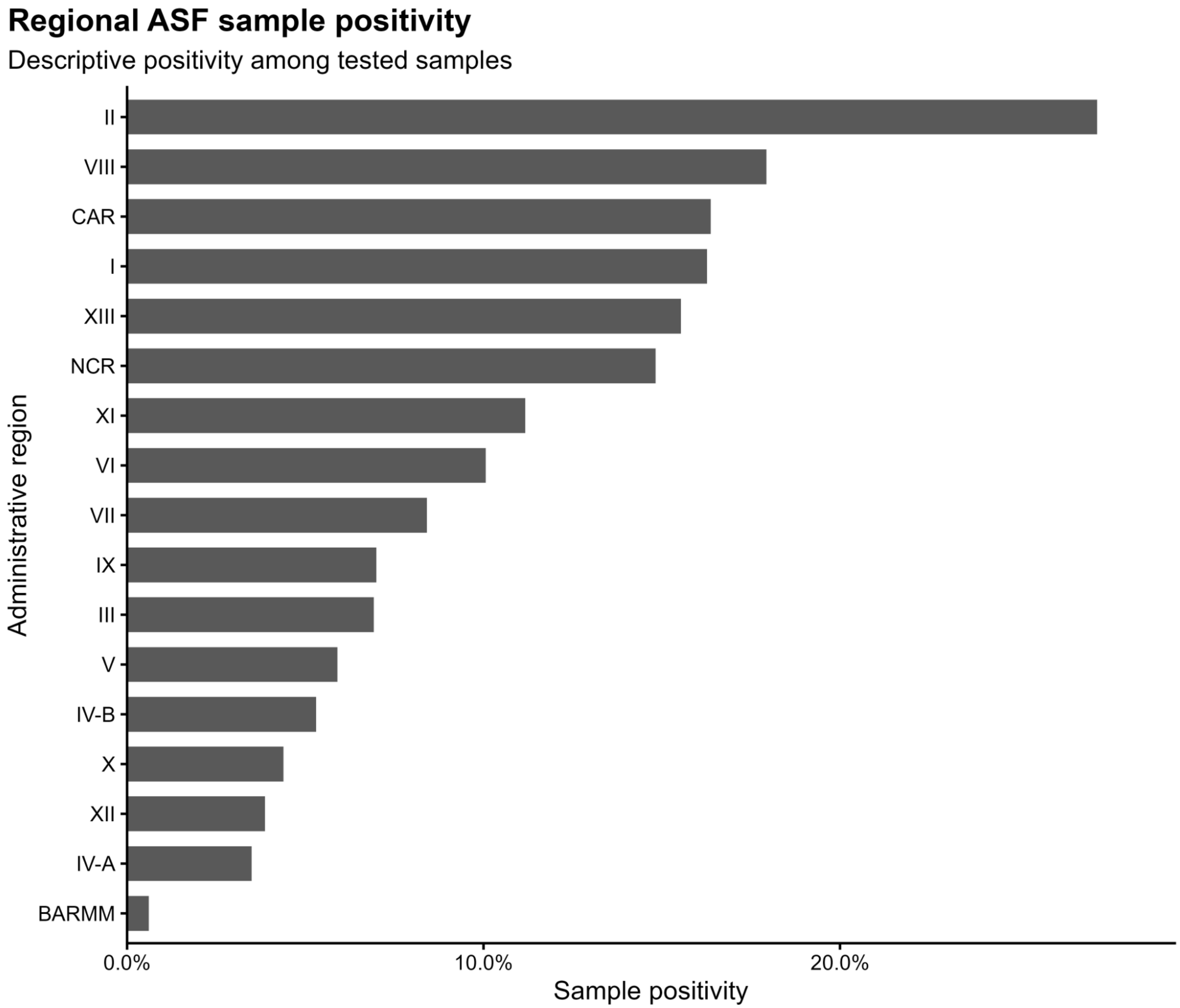

Supplementary Figure S3. Descriptive ASF sample positivity by administrative region.

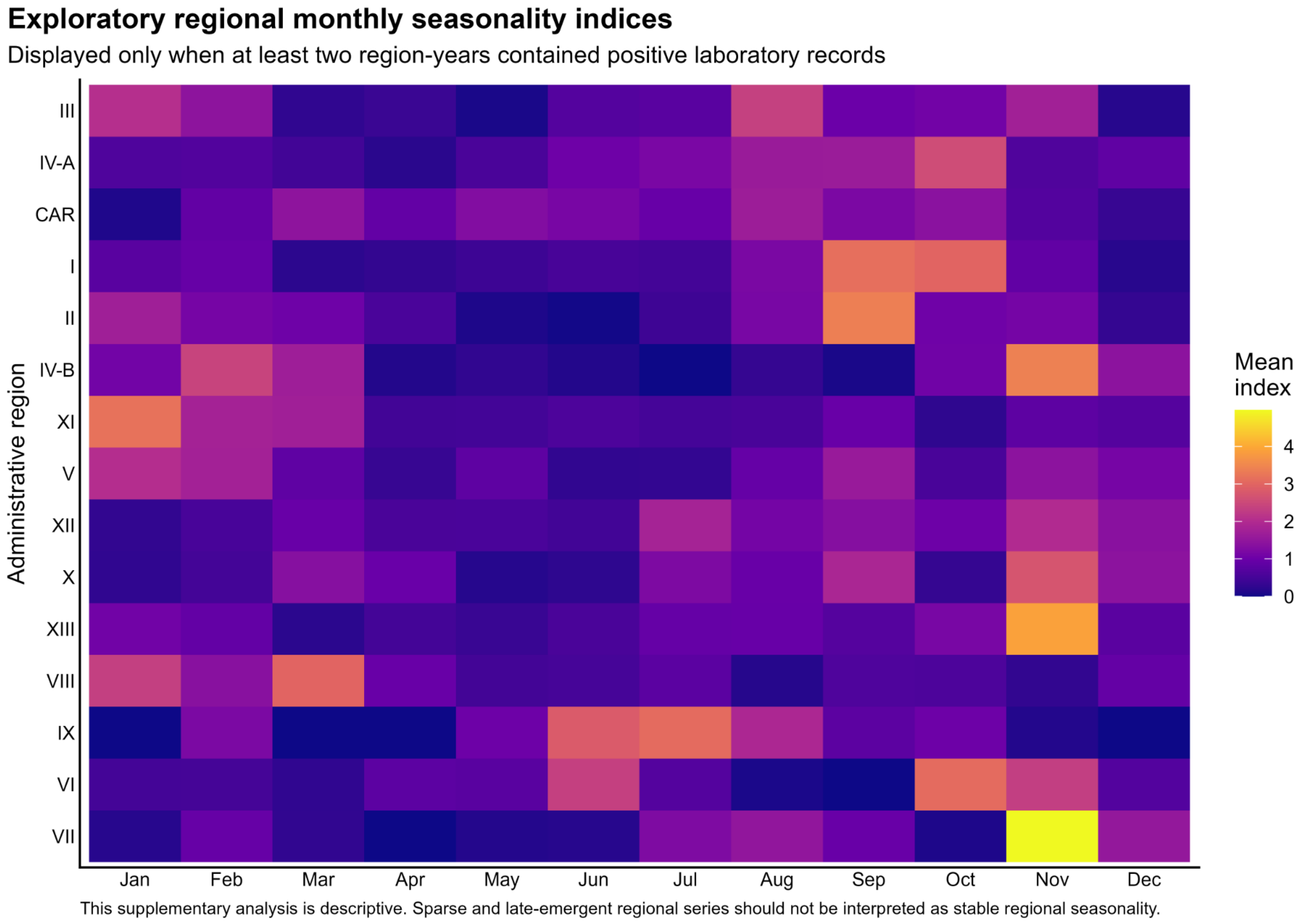

Supplementary Figure S4. Exploratory regional monthly seasonality indices. Cells are displayed when at least two region-years contributed. Sparse and late-emergent regional series should not be interpreted as stable regional seasonality.

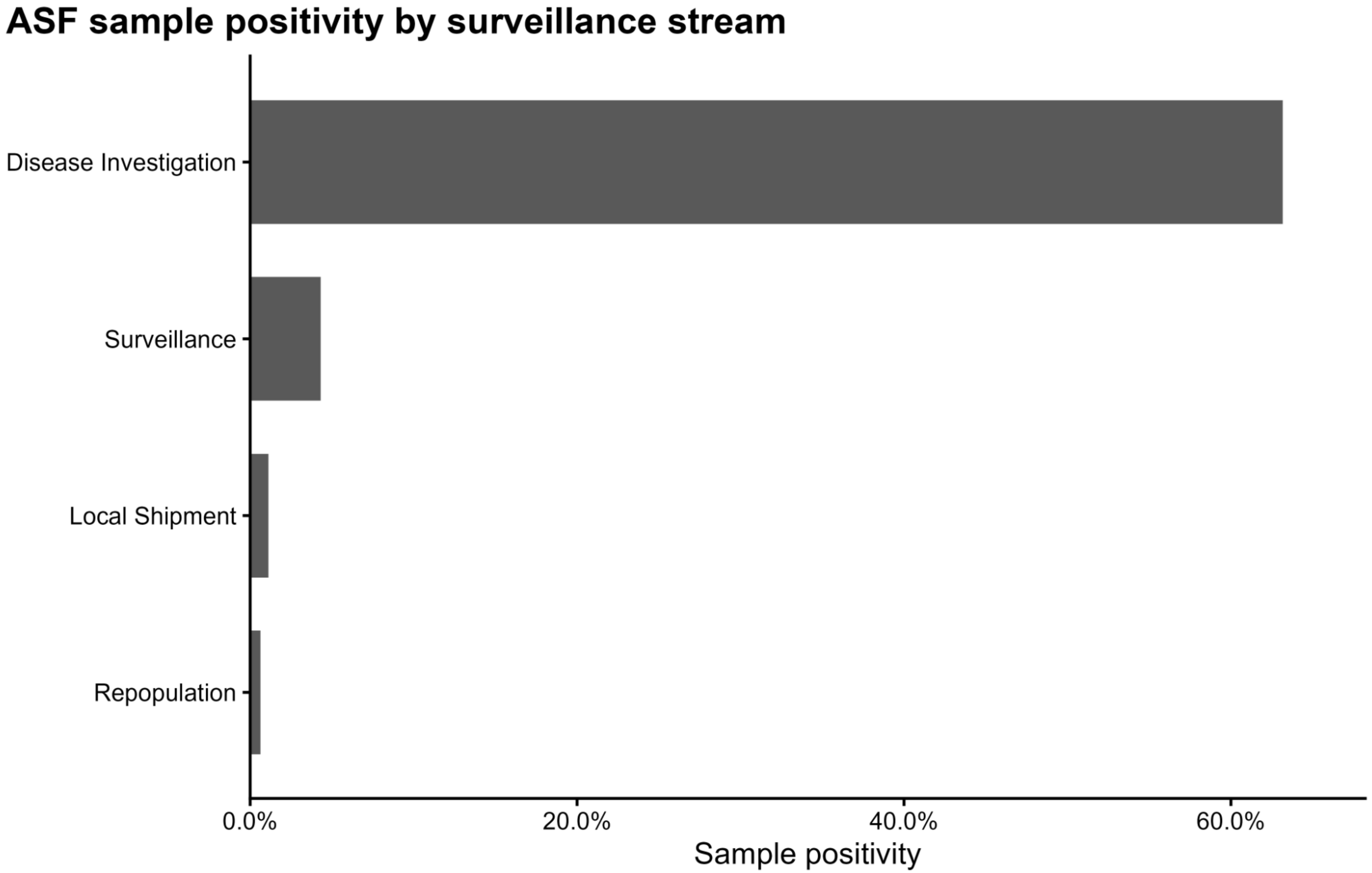

Supplementary Figure S5. ASF sample positivity by surveillance stream.

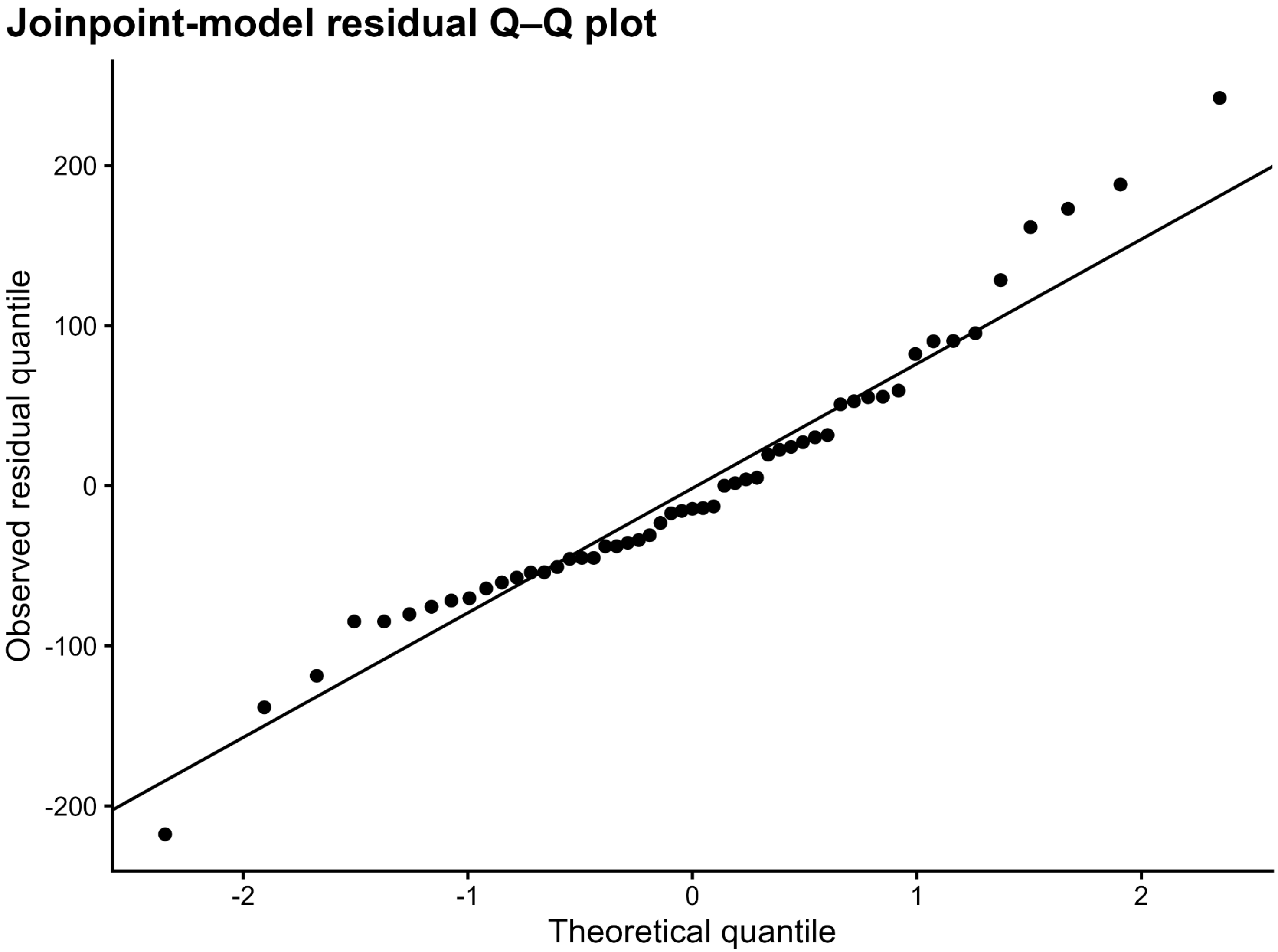

Supplementary Figure S6. Normal Q–Q plot of residuals from the selected joinpoint model.

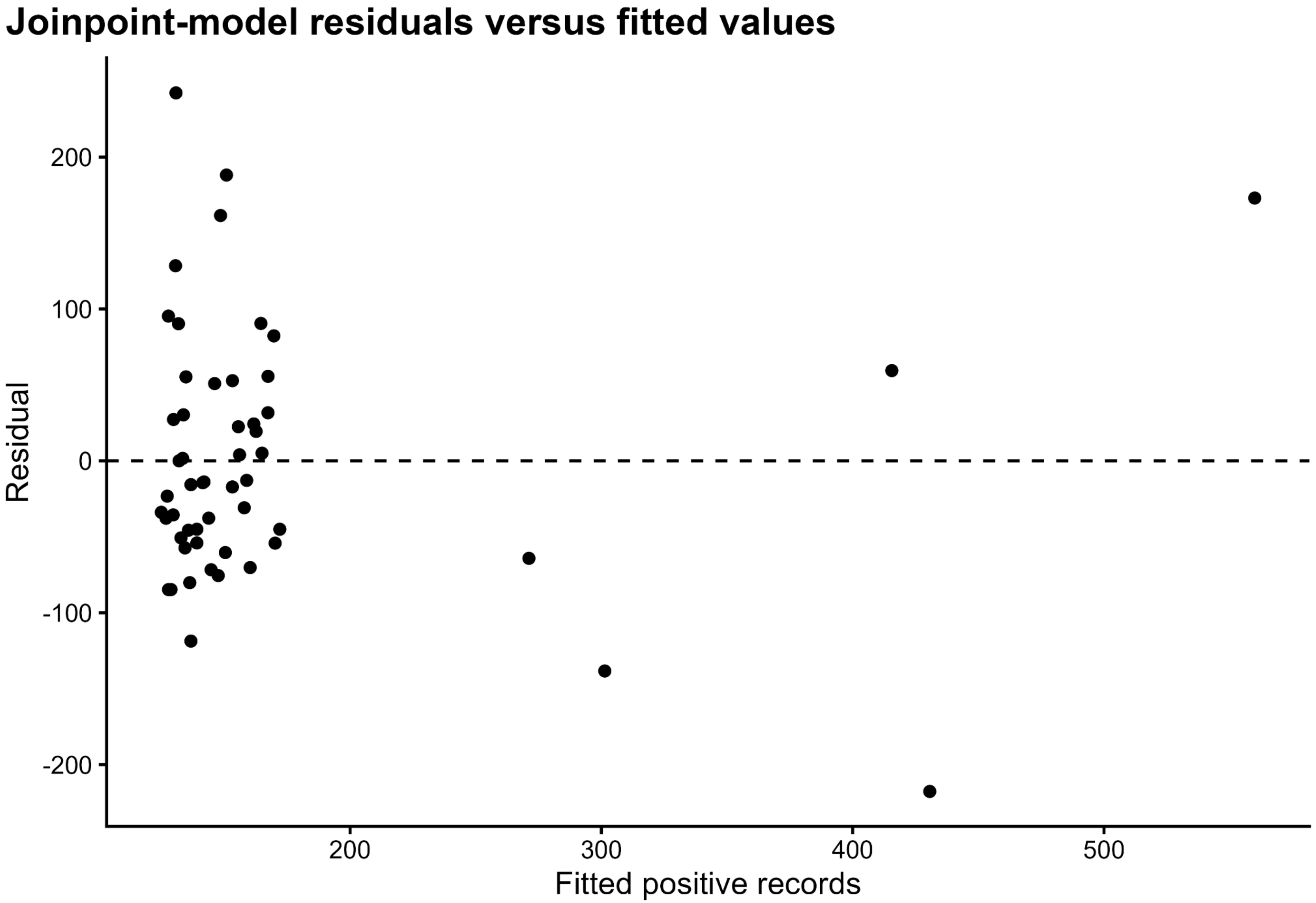

Supplementary Figure S7. Residuals versus fitted values for the selected joinpoint model.

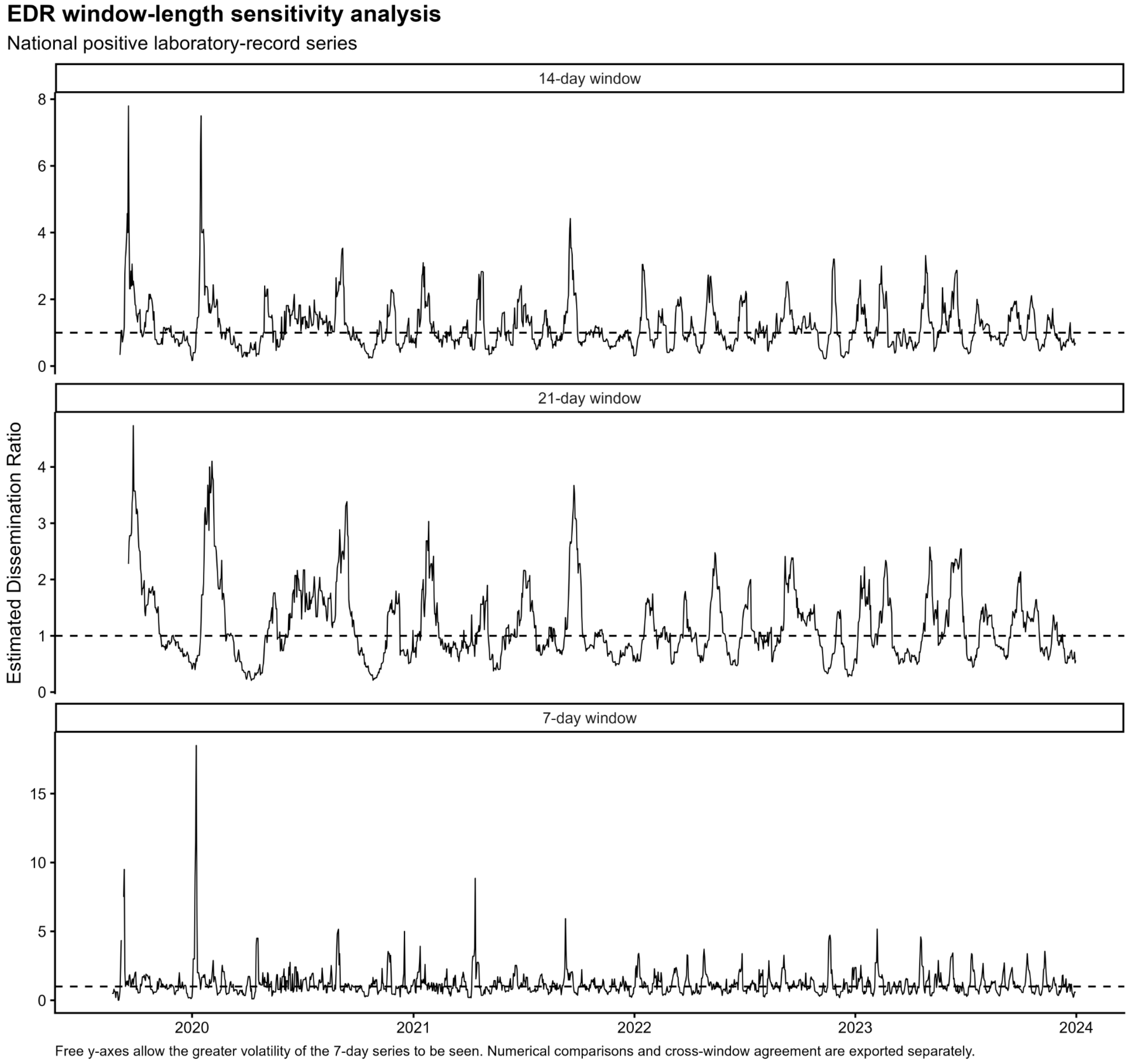

Supplementary Figure S8. Sensitivity of the Estimated Dissemination Ratio to 7-, 14-, and 21-day windows. Free y-axes emphasize the greater volatility of the 7-day specification.

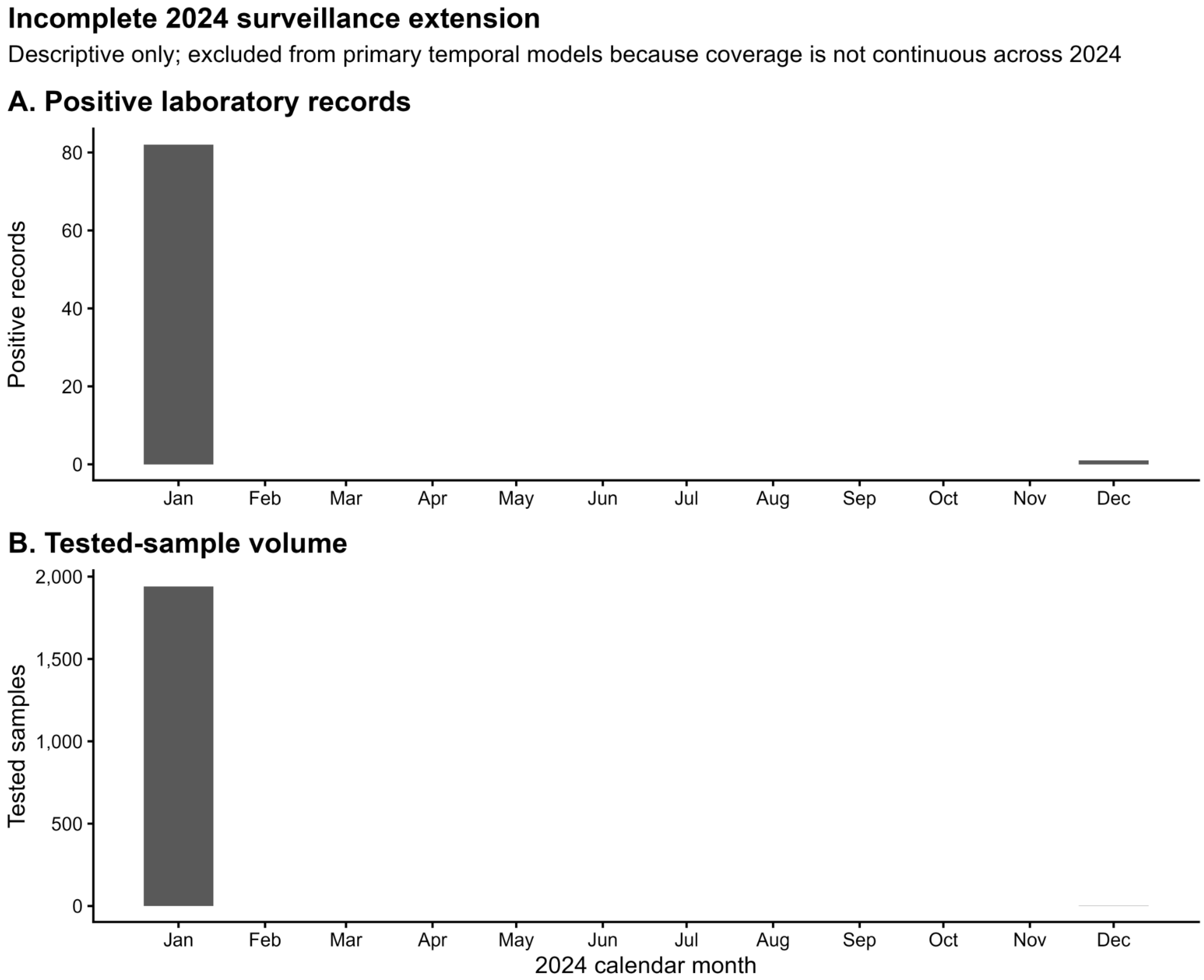

Supplementary Figure S9. Incomplete 2024 surveillance extension. The 2024 records are descriptive only and were excluded from primary temporal models because coverage was concentrated in January with one December record.

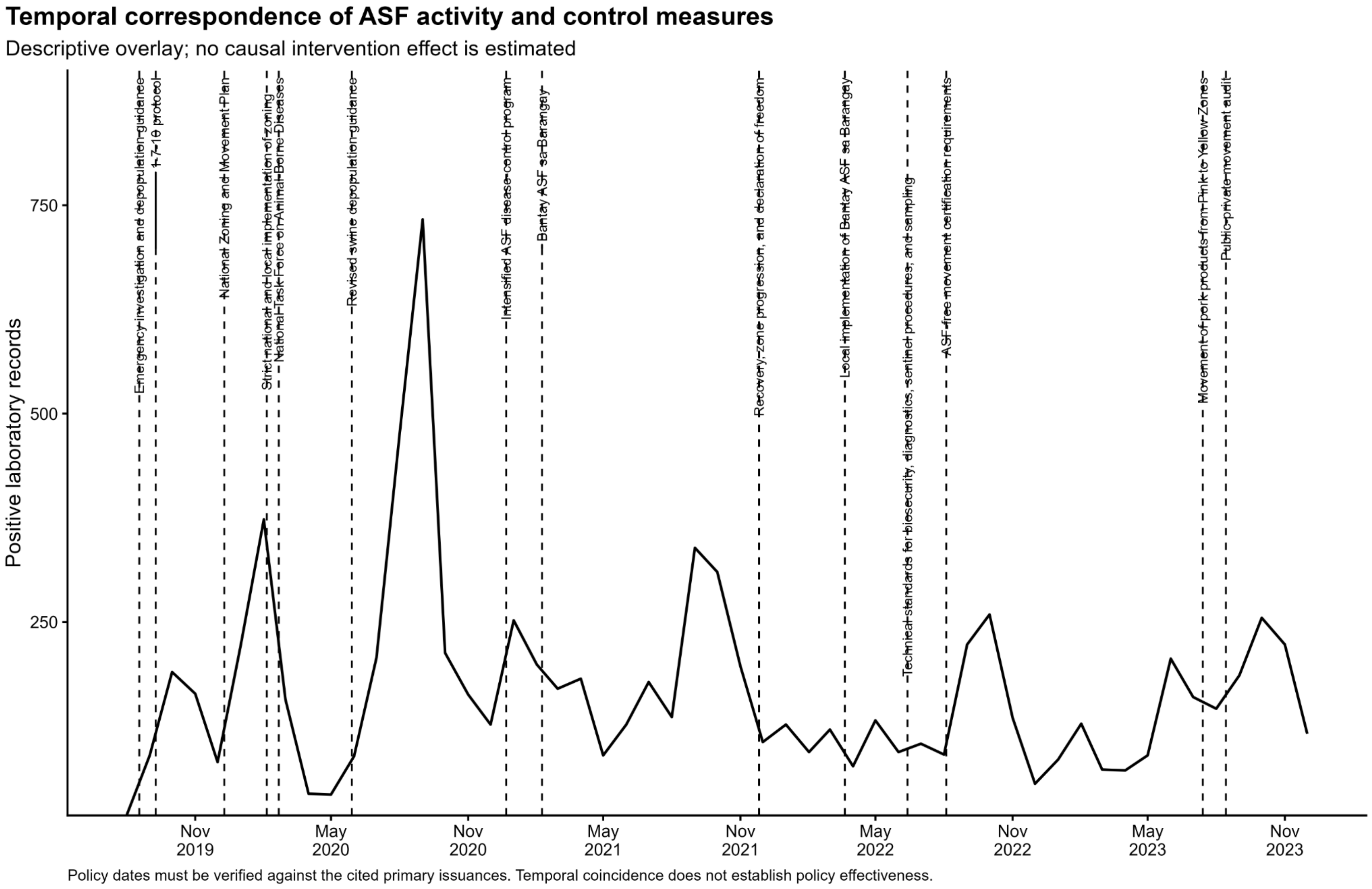

Supplementary Figure S10. Temporal correspondence of monthly ASF-positive laboratory records and major national control measures. The overlay is descriptive; temporal coincidence does not establish policy effectiveness.
